# Identification of novel susceptibility loci for non‐syndromic cleft lip with or without cleft palate

**DOI:** 10.1111/jcmm.15878

**Published:** 2020-10-27

**Authors:** Lan Ma, Shu Lou, Ziyue Miao, Siyue Yao, Xin Yu, Shiyi Kan, Guirong Zhu, Fan Yang, Chi Zhang, Weibing Zhang, Meilin Wang, Lin Wang, Yongchu Pan

**Affiliations:** ^1^ Jiangsu Key Laboratory of Oral Diseases Nanjing Medical University Nanjing China; ^2^ Department of Environmental Genomics Jiangsu Key Laboratory of Cancer Biomarkers, Prevention and Treatment Collaborative Innovation Center for Cancer Personalized Medicine School of Public Health Nanjing Medical University Nanjing China; ^3^ Department of Genetic Toxicology The Key Laboratory of Modern Toxicology of Ministry of Education School of Public Health Nanjing Medical University Nanjing China; ^4^ Department of Orthodontics Affiliated Hospital of Stomatology Nanjing Medical University Nanjing China; ^5^ State Key Laboratory of Reproductive Medicine Nanjing Medical University Nanjing China

**Keywords:** association signals, molecular genetics, orofacial clefts, susceptibility, zebrafish

## Abstract

Although several genome‐wide association studies (GWAS) of non‐syndromic cleft lip with or without cleft palate (NSCL/P) have been reported, more novel association signals are remained to be exploited. Here, we performed an in‐depth analysis of our previously published Chinese GWAS cohort study with replication in an extra dbGaP case‐parent trios and another in‐house Nanjing cohort, and finally identified five novel significant association signals (rs11119445: 3’ of *SERTAD4*, *P* = 6.44 × 10^−14^; rs227227 and rs12561877: intron of *SYT14*, *P* = 5.02 × 10^−13^ and 2.80 × 10^−11^, respectively; rs643118: intron of *TRAF3IP3*, *P* = 4.45 × 10^−6^; rs2095293: intron of *NR6A1*, *P* = 2.98 × 10^−5^). The mean (standard deviation) of the weighted genetic risk score (wGRS) from these SNPs was 1.83 (0.65) for NSCL/P cases and 1.58 (0.68) for controls, respectively (*P* = 2.67 × 10^−16^). Rs643118 was identified as a shared susceptible factor of NSCL/P among Asians and Europeans, while rs227227 may contribute to the risk of NSCL/P as well as NSCPO. In addition, sertad4 knockdown zebrafish models resulted in down‐regulation of sox2 and caused oedema around the heart and mandibular deficiency, compared with control embryos. Taken together, this study has improved our understanding of the genetic susceptibility to NSCL/P and provided further clues to its aetiology in the Chinese population.

## 1 INTRODUCTION

Orofacial clefts are among the most common craniofacial birth defects worldwide with an overall prevalence of one per 700 live births.^1^, ^2^ Affected individuals face feeding difficulties and typically require multiple repair surgeries, therapeutic dental procedures and speech therapy throughout childhood.^3^ Approximately 70% of orofacial clefts are non‐syndromic orofacial clefts (NSOCs), which are commonly categorized as non‐syndromic cleft lip with or without cleft palate (NSCL/P) and non‐syndromic cleft palate only (NSCPO), due to the different developmental origins of the lip and palate.^4^ The aetiology of NSCL/P is related to both genetic susceptibility and epidemiological risk factors such as maternal smoking and alcohol consumption.^5^, ^6^, ^7^, ^8^

In the past few years, genome‐wide association studies (GWAS) have successfully identified thousands of loci associated with complex traits and diseases, including NSCL/P. To date, more than forty susceptibility loci have been reported to be associated with NSCL/P risk, which aid in understanding biological mechanisms involved in the risk of NSCL/P.^9^, ^10^, ^11^, ^12^, ^13^, ^14^ In our previous work, we conducted a case‐control–based GWAS followed by two rounds of replication and identified five genome‐wide significant common variant signals that influence the risk of NSCL/P.^13^

However, the currently confirmed NSCL/P risk loci explain only a fraction of the heritability of NSCL/P. The extent of genetic contribution, including that attributable to common variants, remains largely unknown. One of the reasons is that the threshold of GWAS is very strict, which leads to high false negative. Therefore, studies focusing on SNPs with relatively moderate *P* values of GWAS were demonstrated to be helpful and useful in improving the understanding of the missing heritability of GWAS. For instance, by concentrating on the SNPs with relatively moderate *P* values in the GWAS, Lin *et al* identified additional loci by testing promising associations in an extended 3‐stage validation consisting of 6053 coronary heart disease (CHD) cases and 7410 controls.^15^ Wang *et al* selected 16 significant but unreplicated SNPs from stage 1 of a GWAS analysis to validate their association with the risk of NSCL/P and identified an independent locus in 10q25.3 that was associated with NSCL/P.^16^ Furthermore, the database of Genotypes and Phenotypes (dbGaP) is a highly utilized tool for sharing individual‐level data and summary‐level data from GWAS, sequencing studies and other large‐scale genomic studies,^17^ which was widely used in a large number of researches to increase the understanding of the genetic architecture.^11^, ^12^, ^18^

In the current study, to explore additional promising signals from our previously published Chinese GWAS cohort study, we performed an in‐depth analysis of data from that study, focusing on the risk loci with *P* values ranging from 10^−3^ to 10^−5^ that did not reach genome‐wide significance in the previous GWAS, and then followed by two replications in additional dbGaP case‐parent trios and an in‐house case‐control cohort. We identified five novel significant association signals for NSCL/P. Among them, rs11119445, rs227227 and rs12561877 reached the genome‐wide significance and rs643118 was a shared NSCL/P susceptibility variant between Asian and European populations. Then, we calculated the weighted genetic risk score (wGRS) of the susceptibility loci based on odds ratio of each variant from the replication cohort to assess the predictive ability. Furthermore, morphological defects in embryos were analysed to reveal the potential functional role of genes during zebrafish embryogenesis.

## 2 MATERIALS AND METHODS

### 2.1 Primary GWAS data

As shown in Table 1 and previously reported,^13^ the primary GWAS data consisted of two independent cohorts which were respectively derived from Huaxi (504 NSCL/P cases and 455 newborn controls) and Nanjing (354 NSCL/P cases and 793 controls). All samples from the Huaxi cohort and NSCL/P cases from the Nanjing cohort were genotyped using Affymetrix Axiom Genome‐Wide CHB1 and CHB2 arrays by the CapitalBio corporation (1,280,786 single nucleotide polymorphisms, SNPs); the controls from the Nanjing cohort were from a previous study ^15^ and were genotyped using an Affymetrix Genome‐Wide Human SNP Array 6.0 (905,119 SNPs). To allow for the combination of data derived from different genotyping platforms and to improve coverage of the genome, we used imputed data as a control for the Nanjing cohort. The principal component analysis (PCA) in discovery stage indicated that the cases and controls were genetically matched, without evidence of gross population stratification, which has been described previously.^13^ After the basic quality control, we extracted best‐guess genotype data for SNPs with imputation quality info >0.8 and minor allele frequency (MAF)>0.05 of sex‐matched individuals and combined them with the genotype data of the Nanjing cases.^13^

**Table 1:** Demographic characteristics in NSCL/P cases and controls

Variables	Discovery				Replication		
	Huaxi GWAS data		Nanjing GWAS data		First‐stage dbGaP Asian	Second‐stage In‐house Nanjing cohort	
	Cases (N = 504) N (%)	Controls (N = 455) N (%)	Cases (N = 354) N (%)	Controls (N = 793) N (%)	Case‐parent trios (N = 944) N (%)	Cases (N = 1,050) N (%)	Controls (N = 919) N (%)
Age (mean ± SD)	1.51 ± 0.51	0.00 ± 0.00	5.98 ± 8.02	59.11 ± 9.68	—	4.58 ± 6.88	10.81 ± 2.21
Gender							
Male	308 (61.11)	236 (51.87)	235 (66.38)	565 (71.25)	—	680 (64.76)	502 (54.62)
Female	196 (38.89)	219 (48.13)	119 (33.62)	228 (28.75)	—	370 (35.24)	417 (45.38)

Note

GWAS: genome‐wide association studies; dbGaP: the database of Genotypes and Phenotypes.

### 2.2 SNP selection and regional association plotting

We first performed a meta‐analysis on the two primary GWAS cohorts (Huaxi GWAS and Nanjing GWAS), and selected SNPs for the replication based on the following criteria: (a) 1.00 × 10^−5^ < *P*_meta_<1.00 × 10^‐3^, (b) *P*_Huaxi_ < 5.00 × 10^‐2^ and *P*_Nanjing_ < 5.00 × 10^‐2^, (c) clear genotyping clusters and (4) the SNP with the lowest *P* value was selected when multiple SNPs were observed in high linkage disequilibrium (LD) (*r* ^2^ ≥ .5). The chromosomal region was plotted using LocusZoom 1.1 (http://locuszoom.sph.umich.edu/).

### 2.3 Replication samples

The imputed data of the International Consortium to Identify Genes and Interactions Controlling Oral Clefts (944 Asian trios and 825 European trios)^9^ were retrieved online through dbGaP (http://www.ncbi.nlm.nih.gov/gap) under the accession number phs000094.v1.p1., where individuals had been genotyped using the Illumina Human610_Quadv1_B microarray. These case‐parent trios of the dbGaP database came from different populations. Beaty *et al* conducted a PCA on all parents of cases with non‐syndromic oral cleft to document genetic variation in this consortium.^9^ Among those subjects, 944 NSCL/P case‐parent trios of Asian ancestry were selected as the first‐stage replication cohort.

The second‐stage replication samples were an in‐house cohort including 1,050 NSCL/P cases and 919 healthy controls which were derived from three affiliated hospitals of Nanjing Medical University since August 2008. All subjects were genetically unrelated Han Chinese individuals from Jiangsu Province and the surrounding areas. Each individual donated venous blood samples for genomic DNA extraction after providing written informed consent. The study was approved by the Institutional Review Board of Nanjing Medical University (NJMUERC [2008] No. 20).

### 2.4 DNA extraction and genotyping (second‐stage replication)

Genomic DNA from the second‐stage replication cohort was isolated from peripheral blood lymphocytes of all subjects using the conventional phenol‐chloroform method and the Qiagen Blood kit. TaqMan assays (Applied Biosystems) were performed to genotype all the samples. Genotype analysis was performed by investigators blinded to the case/control status. Approximately 5% of the samples were randomly selected for repeated analysis. Further information on the primers and probes is shown in Table S1.

### 2.5 Weighted genetic risk scores

To develop a risk scoring system based on genetic markers and assess their predictive ability, we used five susceptibility SNPs (rs11119445, rs227227, rs12561877, rs643118 and rs2095293) to calculate weighted genetic risk score (wGRS) values in the second‐stage replication. The wGRS was calculated by multiplying the number of risk alleles for each SNP by its weight according to the following formula:

$$\sum_{i=1}^{k}\beta i*SNPi.$$

where *k* is the number of *SNP* replicates in the study, which equals 5; *βi* is the weight of each *SNP*, which is the natural log of the odds ratio for each allele; and *SNPi* is the number of copies of the risk allele (0, 1 or 2).

### 2.6 In silico bioinformatics analysis on SNPs

The newly identified SNPs were annotated for potential regulatory function by HaploReg v4.1. Three‐dimensional (3D) chromatin looping data (http://cbportal.org/3dsnp/) were used to link promising SNPs to their three‐dimensional interacting genes.^19^ Expression quantitative trait loci (eQTL) analysis was conducted on the candidate SNPs using the Genotype‐Tissue Expression (GTEx) project (http://www.gtexportal.org/).

### 2.7 Gene expression during mouse craniofacial development and human dental pulp stem cell cultures (DPSCs)

Gene expression during growth and fusion of the facial prominences in the C57BL/6J mouse strain during embryonic days (E) 10.5‐14.5 were downloaded from the GEO data set (GSE67985).^20^ Processed microarray expression data from dental pulp stem cell cultures (DPSCs) of NSCL/P patients (N = 7) and controls (N = 6) were searched from EMBL‐EBI (E‐GEOD‐42589) to assess differences in expression levels for the associated genes.^21^

### 2.8 Microinjection of morpholino oligos

The translation‐blocking morpholino antisense oligonucleotide (MO) against sertad4 and standard control MO were synthesized by Gene Tools (Philomath, USA) and injected into in one‐cell stage zebrafish embryos. To control for possible non‐specific effects of MO injection, MOs were injected in p53 mutant zebrafish embryos to check for p53‐induced apoptosis. The sequences of zebrafish control MOs and translation‐blocking sertad4 MOs are listed below: standard control MO, 5.‐ CCTCTTACCTCAGTTACAATTTATA‐3., translation‐blocking sertad4 MOs, 5.‐TCATTGATAAGACCAGAGCCATGCT‐3. 8 ng MO per injection was used in all the experiments.

### 2.9 CRISPR/Cas9‐mediated genome editing (crispant) of sertad4 in zebrafish embryos

The zebrafish *sertad4* gene sequences were obtained from the zebrafish information network (www.zfin.org). Single guide RNAs (sgRNAs) were designed using the CRISPRscan algorithm ^22^ and synthesized by in vitro transcription using MEGAscript™ T7 Transcription Kit (Invitrogen). sgRNAs targeting sertad4 were purified by ethanol precipitation and resuspended in RNAse‐free water. For the CRISPR/Cas9 microinjection, Cas9 protein (GenScript, Z03388‐100) and sgRNA mix were prepared and zebrafish embryos were injected directly with 200 ng/μL and 100 ng/μL of Cas9 and sgRNA per embryo, respectively. To confirm genome editing, direct sequencing of PCR products was applied.

### 2.10 Plasmid construction

The coding sequence (CDS) of human and zebrafish *nr6a1* was amplified and cloned into pXT7‐*NR6A1*‐Human and pXT7‐*nr6a1a*‐Zebrafish between the restriction enzyme site EcoRI and XhoI, respectively. All constructs were confirmed by Sanger sequencing. For the over‐expression experiment, 50 pg human *NR6A1* or zebrafish *nr6a1* mRNAs per embryo was injected into one‐cell stage embryos.

### 2.11 Western blot

Whole‐body tissue from zebrafish embryos at 96h post‐fertilization (hpf) was collected and lysed in RIPA buffer on ice. Western blot was performed as described previously ^23^ and probed with sox2 antibody (diluted 1:5000) (GeneTex, #GT1876) or beta‐actin antibody (diluted 1:1000) (GeneTex, #GT5512).

### 2.12 Statistical analyses

During the discovery stage, we used a fixed‐effects inverse variance method when there was no indication of heterogeneity; otherwise, we adopted a random‐effect model for the corresponding SNPs. The *P* value for heterogeneity was calculated using Cochran's Q, and the proportion of the total variation was quantified by I^2^ statistic. In the replication stage, the association between each SNP and NSCL/P risk in case‐parent trios was evaluated via the transmission disequilibrium test (TDT). The demographic characteristics of cases and controls were analysed by the chi‐squared (χ^2^) test. Hardy‐Weinberg equilibrium (HWE) of genotype frequencies in the control group was tested by Fisher's exact test. Odds ratios (ORs) and 95% confidence intervals (CIs) for the risk of NSCL/P in case‐control studies were assessed in an additive model using logistic regression analyses. For the combined analysis, the case‐control studies and TDT can be jointly analysed by weighted odds ratio to estimate our study‐wide association results.^24^ Conditional analysis was performed to clarify the independent NSCL/P susceptibility signals. Haploview v4.2 software ^25^ was performed for the evaluation of the LD pattern of significant SNPs. Calculating by Power and Sample Size software, we achieved 93.6% and 99.7% power to ensure our results based on the current sample size in discovery stage and second‐stage replication respectively. Data analysis was performed by PLINK 1.90 or R 3.5.3.

## 3 RESULTS

### 3.1 Study overview

**Figure 1:**
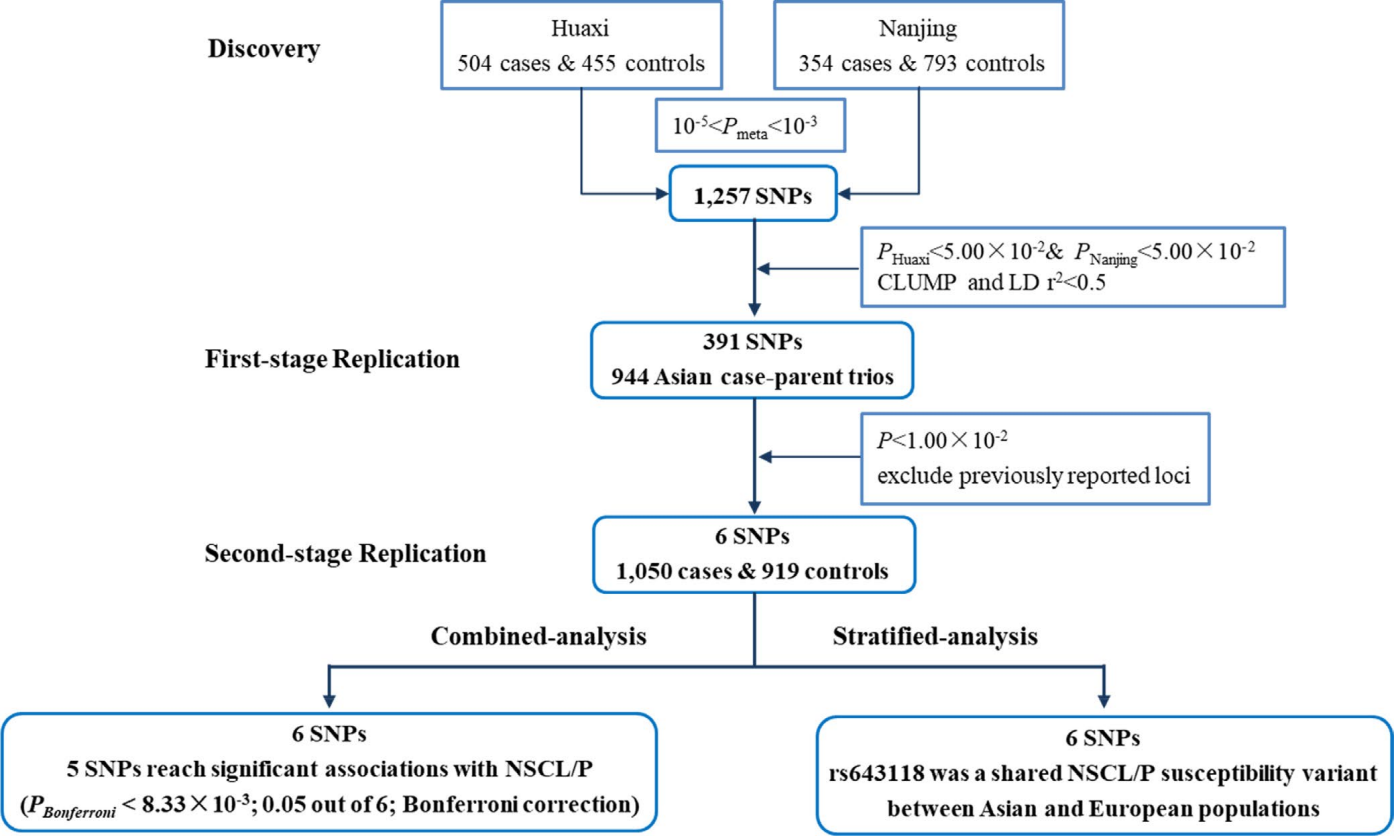
Flow chart of this study

To discover additional susceptibility variants for NSCL/P in the Chinese population, we first conducted a meta‐analysis of two previously published GWAS, totalling 2,106 individuals from the Chinese population and 842,556 genetic variants that passed quality control (Table 1 and Figure 1). Then, based on the selection criteria (see Materials and Methods), a total of 391 SNPs were chosen for replication (Table S2) in the Asian group of the dbGaP database, and 6 SNPs were selected and further replicated in an independent cohort of 1,050 cases and 919 controls.

### 3.2 Combined analysis identified susceptibility loci for NSCL/P

A combined analysis in all stages was performed to assess the comprehensive effects of these susceptible loci. The results showed that five genetic variants were associated with NSCL/P risk (*P*_Bonferroni_ = 8.33 × 10^−3^; 0.05/6): rs11119445, rs227227, rs12561877, rs643118 and rs2095293, of which rs11119445, rs227227 and rs12561877 reached the genome‐wide significance (Table 2). None of the associated variants detected by this study were in high LD (r^2^ ≥ 0.5) with each other or the published NSCL/P risk variants.

**Table 2:** Summary associations between the six genetic variants and risk of NSCL/P in different stages among Asians

SNP	Chr	Gene Neighbourhood	Position	Alleles	Discovery stage			First‐stage replication			Second‐stage replication			Combined	
					(Huaxi and Nanjing GWAS)			(dbGaP Asian)			(In‐house Nanjing cohort)			(All stages)	
					MAF_huaxi_ (control/case)	OR (95% CI)	*P*	MAF (control/case)	OR (95% CI)	*P*	MAF (control/case)	OR (95% CI)	*P*	OR (95% CI)	*P*
rs11119445	1	*SERTAD4*	210 224 051	G/A	0.449/0.391	0.73 (0.63‐0.84)	1.99E‐05	0.383/0.306	0.74 (0.63‐0.86)	5.87E‐05	0.406/0.302	0.64 (0.56‐0.73)	2.43E‐11	**0.73 (0.67‐0.79)**	**6.44E‐14**
rs227227	1	*SYT14*	210 021 655	T/C	0.409/0.469	1.39 (1.20‐1.60)	1.03E‐05	0.452/0.460	1.48 (1.28‐1.71)	7.26E‐08	0.409/0.499	1.44 (1.27‐1.64)	1.92E‐08	**1.34 (1.24‐1.45)**	**5.02E‐13**
rs12561877	1	*SYT14*	210 042 069	C/T	0.269/0.221	0.71 (0.60‐0.84)	1.46E‐04	0.229/0.222	0.68 (0.57‐0.82)	2.45E‐05	0.254/0.179	0.63 (0.54‐0.74)	1.28E‐08	**0.72 (0.65‐0.79)**	**2.80E‐11**
rs643118	1	*TRAF3IP3*	209 761 496	C/T	0.229/0.270	1.36 (1.15‐1.60)	3.00E‐04	0.240/0.308	1.26 (1.06‐1.49)	7.63E‐03	0.198/0.238	1.28 (1.10‐1.49)	1.90E‐03	**1.25 (1.13‐1.37)**	**4.45E‐06**
rs2095293	9	*NR6A1*	124 528 833	C/T	0.230/0.278	1.31 (1.11‐1,54)	7.40E‐04	0.235/0.235	1.34 (1.12‐1.59)	1.12E‐03	0.221/0.255	1.20 (1.03‐1.39)	1.77E‐02	**1.22 (1.11‐1.34)**	**2.98E‐05**
rs1925518	16	*GPR139*	20 132 769	G/T	0.065/0.090	1.57 (1.20‐2.05)	8.11E‐04	0.085/0.027	0.68 (0.52‐0.89)	3.87E‐03	0.154/0.115	0.72 (0.60‐0.86)	4.46E‐04	0.87 (0.68‐1.11)	2.57E‐01

Note

SNP, single nucleotide polymorphism; Chr, chromosome.

Bold values represent significance.

dbGaP, the database of Genotypes and Phenotypes; GWAS, genome‐wide association studies.

* Major/Minor allele.

**Figure 2:**
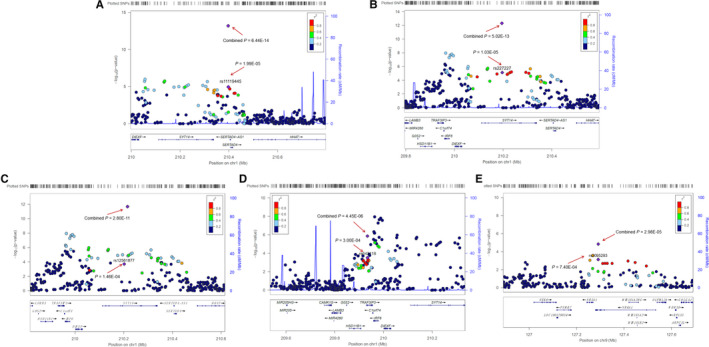
Regional plot of the five newly identified SNPs. The regional plots indicate five variants (A) rs11119445, (B) rs227227, (C) rs12561877, (D) rs643118 and (E) rs2095293 and linkage disequilibrium structure. Logistic regression analyses were used to test the genetic association under an additive model. The marker SNPs are shown as purple diamonds with *P* values in primary GWAS meta and as with their *P* values in the combined stage when all of the NSCL/P cases and controls from the primary GWAS meta and replication I, II were pooled together. Points are colour‐coded based on linkage disequilibrium (*r* ^2^) in Asians. For each plot, the recombination rates (right y‐axes) of the region are shown according to their chromosomal positions (x‐axis)

Rs11119445 (G > A) maps 8.7 kb 3' of *SERTAD4* (*P* = 6.44 × 10^−14^) (Figure 2A). Rs227227 (T > C, *P* = 5.02 × 10^−13^) and rs12561877 (C > T, *P* = 2.80 × 10^−11^) are located in the intron of *SYT14* with LD ranging from 0.2 to 0.4 (Figure 2B,C). In addition, these two SNPs showed independent significant associations with NSCL/P risk under conditioned analysis on each other (*P*_conditional_ = 2.47 × 10^−4^ and 1.80 × 10^−4^ for rs227227 and rs12561877, respectively). We detected another association on 1p32.2 (rs643118 C > T, *P* = 4.45 × 10^−6^) which lies in the intron of *TRAF3*‐interacting protein 3 *(TRAF3IP3)* (Figure 2D). Rs2095293 (C > T) resides in an intron of *NR6A1* (*P* = 2.98 × 10^−5^) on 9q33.3 (Figure 2E).

### 3.3 The prediction value of the identified variants for NSCL/P

The wGRS was based on the ORs reported for the cumulative effect of multiple genetic risk variants, which were calculated from the data of the 1,050 cases and 919 controls in the second‐stage replication. The mean (standard deviation) of wGRS was 1.83 (0.65) in the NSCL/P cases and 1.58 (0.68) in controls, respectively, which showed a clear separation of the scores between the cases and controls (*P* = 2.67 × 10^−16^) (Figure S1). Ascertainment of those with high wGRS values may provide a theoretical basis for prevention.

### 3.4 Associations between SNPs and risk of NSCL/P across racial groups

**Figure 3:**
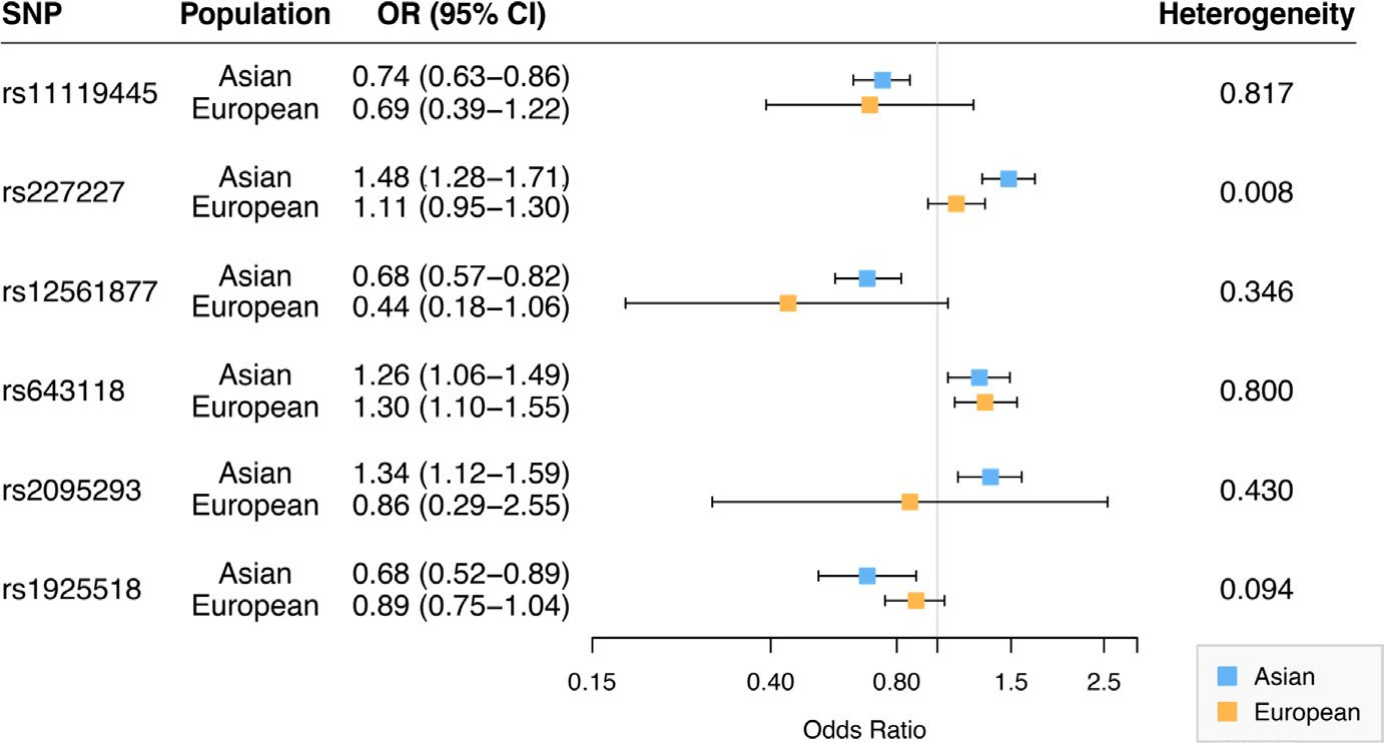
Comparison of associations for the six genetic variants in Asian and European ancestry populations. *P*_heterogeneity_ was calculated to test the difference in the association of each of the SNPs with risk of NSCL/P across these two populations

We further evaluated the above six genetic variants in NSCL/P case‐parent trios in the dbGaP database across racial groups. Rs643118 was significantly associated with an increased risk of NSCL/P in Europeans (*P* = 2.76 × 10^−3^), which was consistent with the findings in the Asian populations. However, the other five NSCL/P risk SNPs selected in the Asian group of the dbGaP database were not replicated in populations of European ancestry (Figure 3).

### 3.5 Annotation and functional assessment of genetic variants

As predicted by HaploReg v4.1, rs11119445 and rs12561877 have regulatory effects on the transcriptional enhancer factor‐1 (TEF‐1) motifs (Table S3). The 3D chromatin looping data in blood demonstrated that the interacting genes of rs11119445 are *SERTAD4* and *SERTAD4‐AS1* (Figure S2). We conducted eQTL analysis based on the GTEx database and found that rs643118 exhibited a significant association with the expression of *TRAF3IP3* (*P* = 1.60 × 10^−12^) in whole blood (Figure S3).

### 3.6 Gene expression in mouse craniofacial structures and human DPSCs

We examined the gene expression in the proximal and distal maxilla of mice during embryonic E10.5‐E14.5 period (Figure S4). The expression levels of the genes (*Sertad4, Syt14, Traf3ip3, Nr6a1*) vary greatly over that period. Comparison of *Nr6a1* expression in the proximal and distal parts of the maxilla and mandible showed different expression patterns relative to the other three genes. Then, we compared microarray expression data from DPSCs of NSCL/P cases (n = 7) and controls (n = 6) and found that *SERTAD4* was significantly down‐regulated (*P* = .039), while *NR6A1* was significantly up‐regulated in the DPSCs of NSCL/P patients (*P* = .008, Figure S5).

### 3.7 Effects of candidate genes in zebrafish embryo models

To explore the functional roles of the relevant genes during zebrafish embryogenesis, the zebrafish embryos after microinjection were collected and imaged with transmitted light using a stereomicroscope at 0h, 24h, 48h, 72h and 96h post‐fertilization (hpf).

**Figure 4:**
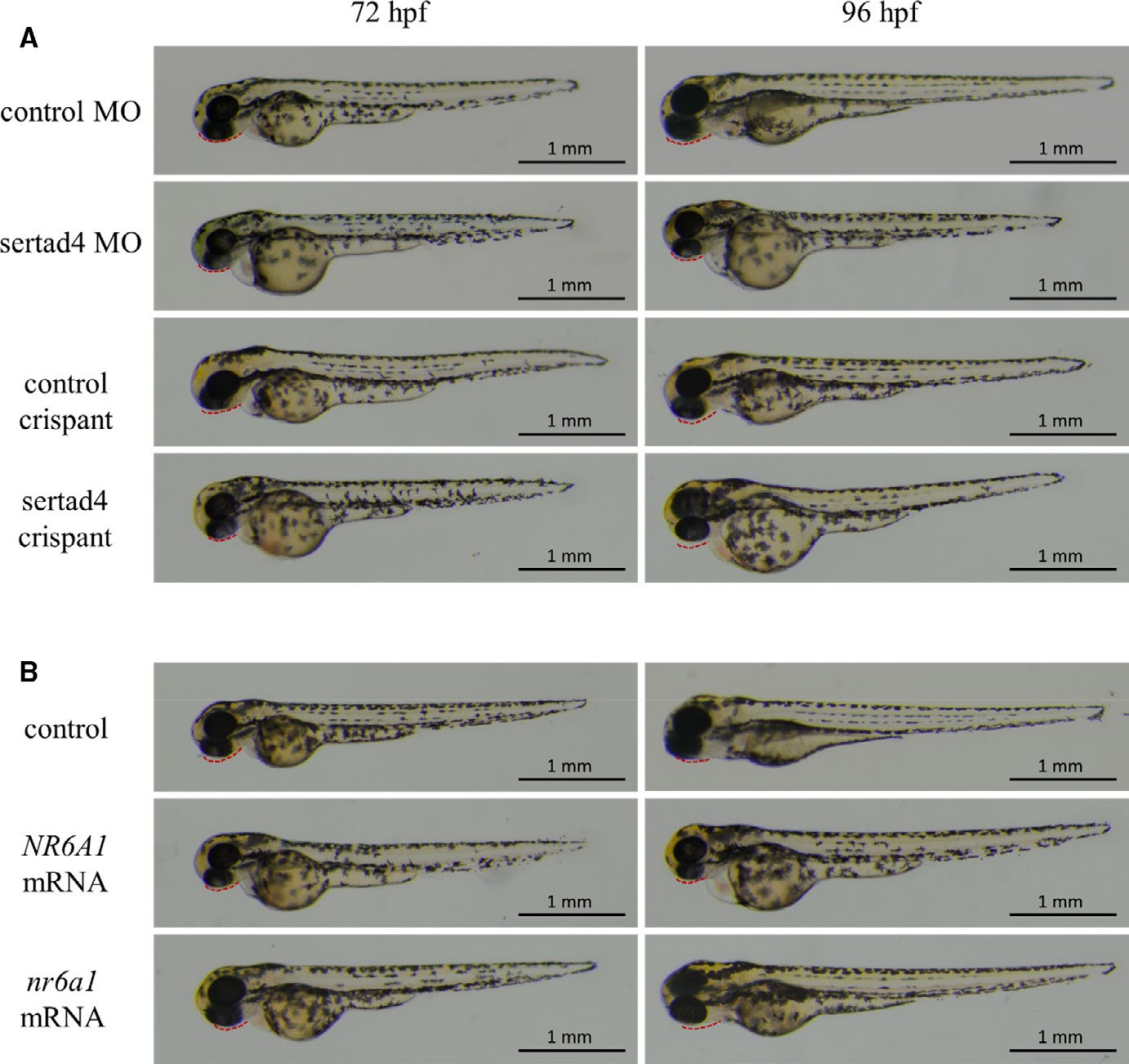
Zebrafish larvae were imaged with transmitted light. (A) Translation‐blocking morpholino and CRISPR/Cas9‐based targeted sertad4 knockdown (crispant) zebrafish models, (B) Embryos with over‐expression of human *NR6A1* or zebrafish *nr6a1a*. Mandibular indicated by red dotted line

We generated zebrafish models with partial loss of sertad4 function, including translation‐blocking morpholino and CRISPR/Cas9‐based targeted sertad4 knockdown zebrafish models and investigated the morphological defects in different embryonic stages. Both sertad4 MO and crispant embryos exhibited a shorter body length, mandibular deficiency and oedema around the heart (Figure 4A). Injecting *p53* ^−/−^ embryos with sertad4 MO generated the same morphological abnormalities, indicating that the deformities observed in morphants are not caused by up‐regulation of the p53‐dependent apoptotic pathway ^26^ (Figure S6A). Further, since Sox2 regulates development of the palate rugae, and a loss of this palate signalling centre may contribute to clefting.^27^ Thus, we investigated the expression of sox2 and found it was significantly down‐regulated in sertad4 knockdown zebrafish models at 96hpf (Figure S6B).

However, embryos with over‐expression of human *NR6A1* or zebrafish *nr6a1a* did not exhibit any significant abnormalities (Figure 4B).

## 4 DISCUSSION

The present study investigated the additional promising signals related to NSCL/P based on our previously published Chinese GWAS data. These signals were not replicated in the previous GWAS and were subsequently replicated in extra dbGaP case‐parent trios and case‐control populations. Five novel significant association signals were identified. The wGRS based on the ORs of these signals in the second‐stage replication showed a clear separation of the scores between the cases and controls. Further investigations of the wGRS with other parameters such as environmental risk factors are needed. Interestingly, of the five NSCL/P risk variants, only rs643118 was a susceptibility variant shared between Asian and European populations, suggesting obvious genetic heterogeneity between these two populations. Furthermore, we generated zebrafish models of the candidate genes based on the existing databases to explore the functional roles of the genes during embryogenesis.

The genetic region, 1q32.2, was demonstrated by multiple GWAS to be associated with NSCL/P and NSCPO. The LD between our newly identified SNPs (rs11119445, rs227227, rs12561877 and rs643118) and other SNPs in this region reported to be associated with NSCL/P is low (*r* ^2^ < .5 in 1000 Genomes Project data from an Asian population) (Table S4). Interestingly, rs227227 was in moderate LD with rs2485893 (*r* ^2^ = .6, 10 kb 3’ of *SYT14*) whose G allele was associated with NSCPO among Western Han Chinese,^28^ indicating that rs227227 may be a shared susceptibility factor for NSCL/P and NSCPO among the Chinese population. Together, these results indicate that our finding represents novel significant association signals and illustrates the complex genetic architecture of 1q32.2.

The associated SNPs at the 1q32.2 locus span 8.7 kb 3' of *SERTAD4, SYT14* and *TRAF3IP3*. We evaluated the LD pattern of the significant SNPs at the 1q32.2 and found that almost all of the SNPs that in high LD with them were in these three genes on the Chr 1. Little is known about *SERTAD4* in the craniofacial development, which is a conserved orthologue of the SERTA domain family. Proteins containing the SERTA domain have previously been linked to cell cycle progression and chromatin remodelling.^29^ The sertad4 knockdown in our zebrafish model induced mandibular deficiency, heart failure and down‐regulation of Sox2 protein which had been implicated in various processes of early embryogenesis.^27^, ^30^ *SYT14* participates in pathomechanical neurodegeneration and contributes to abnormal neurodevelopment.^31^ Previous studies found that the expression of *Syt14* was highly restricted to the mouse heart and testis but absent in the brain, suggesting that *Syt14* may be involved in membrane trafficking in specific tissues other than the brain.^32^ RNA‐mediated *SYT14* knockdown can inhibit proliferation and colony formation and promote apoptosis of glioma cells.^33^ *TRAF3IP3* is expressed in the immune system and participates in cell maturation, tissue development and immune response.^34^ It was reported to be one of the network hubs, which suggested a potential role in the head and neck squamous cell carcinoma evolution mechanisms related to inflammation and the microenvironment.^35^

Our study identified a new risk locus at 9q33.3 marked by rs2095293 which lies in the intron of *NR6A1*. The *NR6A1* gene encodes the nuclear receptor subfamily 6 group A member 1. It is expressed at a high level only in the testis and involved in regulating embryonic stem cell differentiation, reproduction and neuronal differentiation.^36^ Increased *NR6A1* was important in maintenance of somitogenesis and posterior development and essential for embryonic survival.^37^, ^38^ The expression level of *NR6A1* was significantly up‐regulated in the DPSCs of NSCL/P cases compared with the controls. Additionally, the expression of *Nr6a1* varies greatly from 10.5 to 14.5 days based on RNA‐seq data of embryonic mouse tissues, suggesting its importance during the development stages of embryos. Furthermore, according to the DECIPHER database, *NR6A1* was also associated with submucous cleft hard palate. However, no significant abnormalities were observed in zebrafish models with over‐expression of human *NR6A1* or zebrafish *nr6a1a*. Further functional evaluations are warranted to explore its roles in the process of orofacial development.

Overall, the combined analysis of two previously published GWAS of NSCL/P with further two‐stage replication has identified five novel significant association signals, including two new risk loci for NSCL/P at 1q32.2 and 9q33.3 in the Chinese population. Ascertainment of those with high wGRS values may provide a theoretical basis for prevention. In the future, additional functional validation studies are warranted to elucidate the aetiology of NSCL/P.

## CONFLICT OF INTEREST

The authors declare no conflicts of interest.

## AUTHOR CONTRIBUTIONS

**lan ma:** Conceptualization (equal); Project administration (equal); Writing‐original draft (equal); Writing‐review & editing (equal). **Shu Lou:** Data curation (equal); Writing‐original draft (equal); Writing‐review & editing (equal). **Ziyue Miao:** Methodology (equal). **Siyue Yao:** Formal analysis (equal). **Xin Yu:** Data curation (equal). **Shiyi Kan:** Formal analysis (equal); Methodology (equal). **Guirong Zhu:** Data curation (equal); Investigation (equal). **Fan Yang:** Supervision (equal); Validation (equal). **Chi Zhang:** Software (equal); Writing‐review & editing (equal). **Wei‐Bing Zhang:** Methodology (equal); Writing‐review & editing (equal). **Meilin Wang:** Data curation (equal); Resources (equal). **Lin Wang:** Resources (equal); Writing‐review & editing (equal). **Yongchu Pan:** Conceptualization (equal); Project administration (equal); Writing‐review & editing (equal).

## Supporting information

Figure S1‐S6

Table S1‐S4
