# Supplementary material for: Identification of novel susceptibility loci for non‐syndromic cleft lip with or without cleft palate
Source: J Cell Mol Med. 2020 Oct 27;24(23):13669–78. doi: 10.1111/jcmm.15878 (PMC7754035; doi:10.1111/jcmm.15878)
Supplement: Supplementary file 1 — Figure S1‐S6 [file JCMM-24-13669-s001.docx]

**Identification of novel susceptibility loci for Nonsyndromic Cleft Lip with or without Cleft Palate**

**Supplementary Information**

**Supplementary Figure 1. Risk for cleft lip/palate according to weighted genetic risk scores.** Weighted genetic risk scores (wGRS) among cases versus controls in Nanjing cohort. Within each boxplot, the horizontal lines reflect the median, the top and bottom of each box reflect the interquartile range, and the whiskers reflect the maximum and minimum values within each grouping.

**Supplementary Figure 2. Chromosome interactions and epigenetic signatures in blood related to rs11119445 in 3DSNP database.** In the circular plot, from outer to inner, the circle represents chromatin states, annotated genes, histone modification set (red), transcription factor set (blue), current SNP and associated SNPs, and 3D chromatin interactions, respectively.

**Supplementary Figure 3. Violin plots for *TRAF3IP3* expression in samples with different genotypes of rs643118 (TT, TC, CC) taken from whole blood.** The plot was generated from the Genotype-Tissue Expression (GTEx) project (http://www.gtexportal.org/home/).

**Supplementary Figure 4.** **Gene expression in the proximal and distal locations of maxilla and mandible during mouse embryonic E10.5-E14.5 period.**

Man.Distal: Mandibular diatal location; Man.Proximal: Mandibular proximal location; Max.Distal: Maxillary distal location; Max.Proximal: Maxillary proximal location. Figures showed expression levels of (a) *SERTAD4* (b) *SYT14* (c) *TRAF3IP3* (d) *NR6A1* in proximal and distal locations of maxilla and mandible during mouse embryonic E10.5-E14.5 stage based on GEO database (GSE67985).

**Supplementary Figure 5**. **Differential gene expression of dental pulp stem cells in NSCL/P patients and normal controls.**

**Supplementary Figure 6. (a) Comparison of the morphology of setrad4 MO injected wild-type (WT) and *p53*^-/-^ larvae at 96 hpf. (b) Sox2 protein expression levels in sertad4 MO or crispant zebrafish embryos at 96 hpf.**


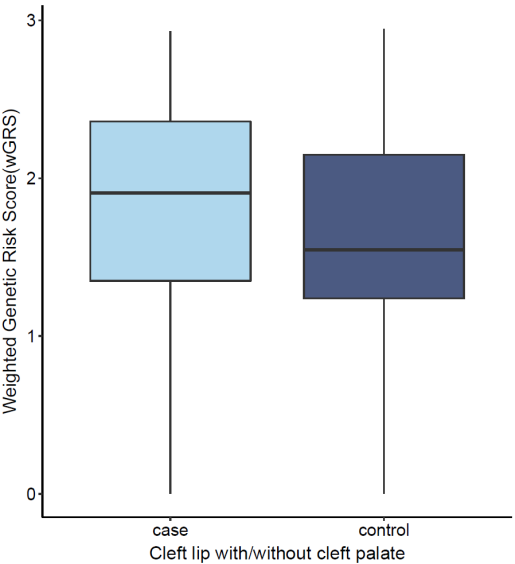


*P*=2.67×10^-16^

**Supplementary Figure 1.** Risk for cleft lip/palate according to weighted genetic risk scores


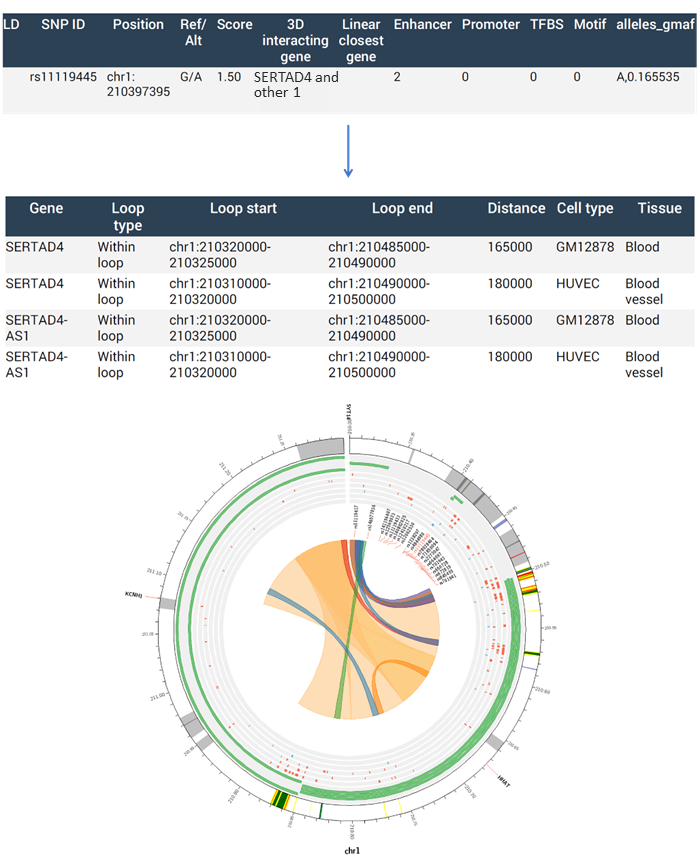


**Supplementary Figure 2**. Chromosome interactions and epigenetic signatures in blood related to rs11119445 in 3DSNP database


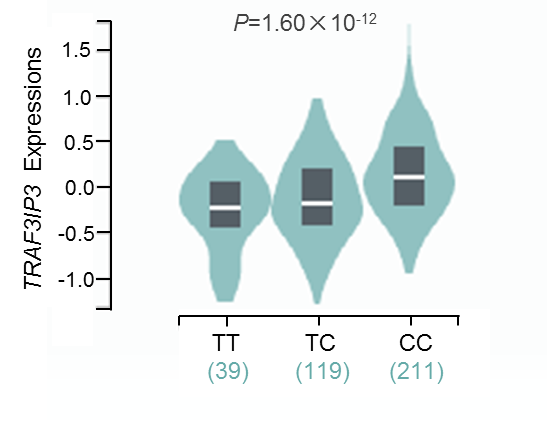


**Supplementary Figure 3.** Violin plots for *TRAF3IP3* expression among whole blood with different genotypes of rs643118 (TT, TC, CC)


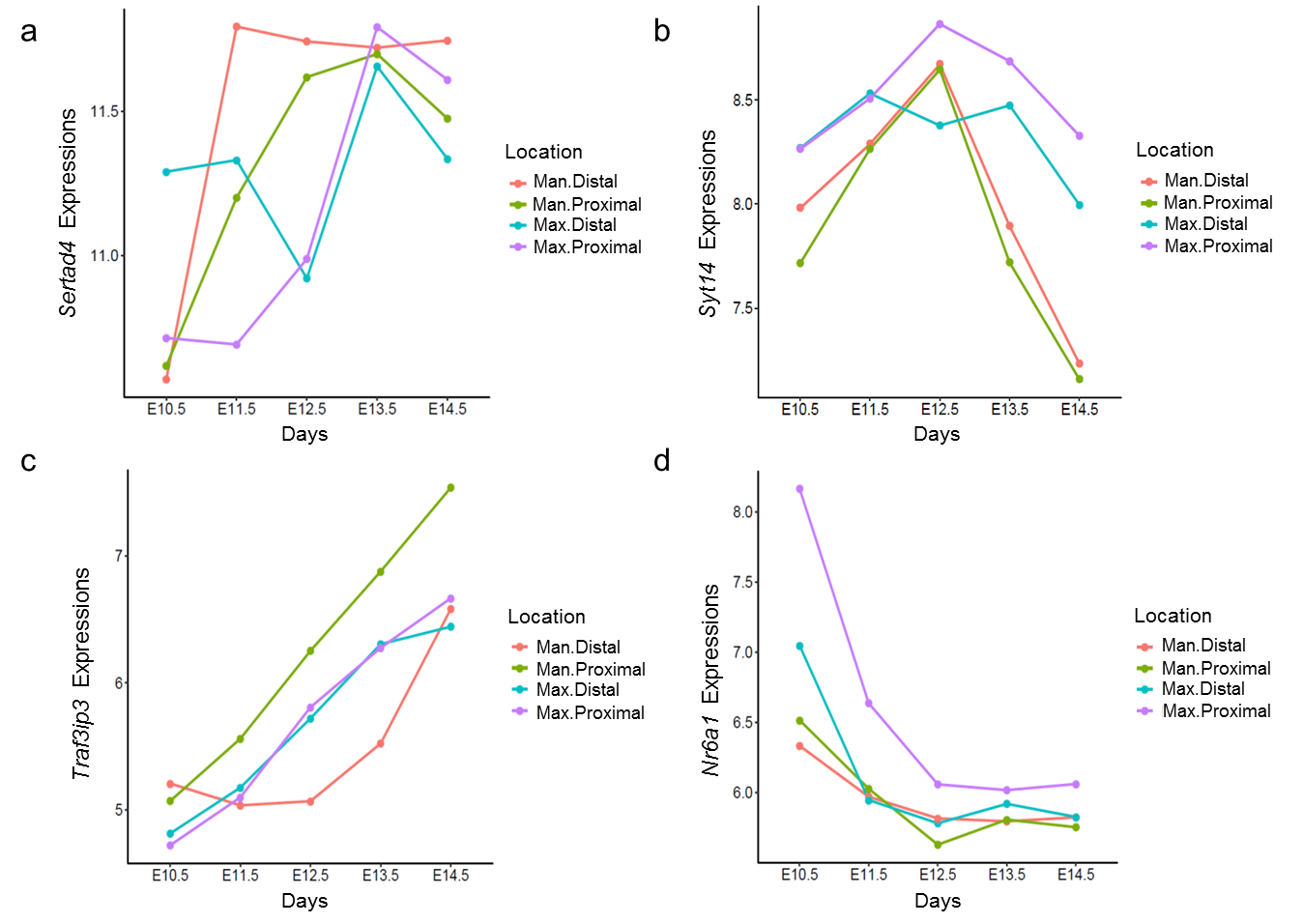


**Supplementary Figure 4**. Gene expression in the proximal and distal locations of maxilla and mandible during mouse embryonic E10.5-E14.5 period


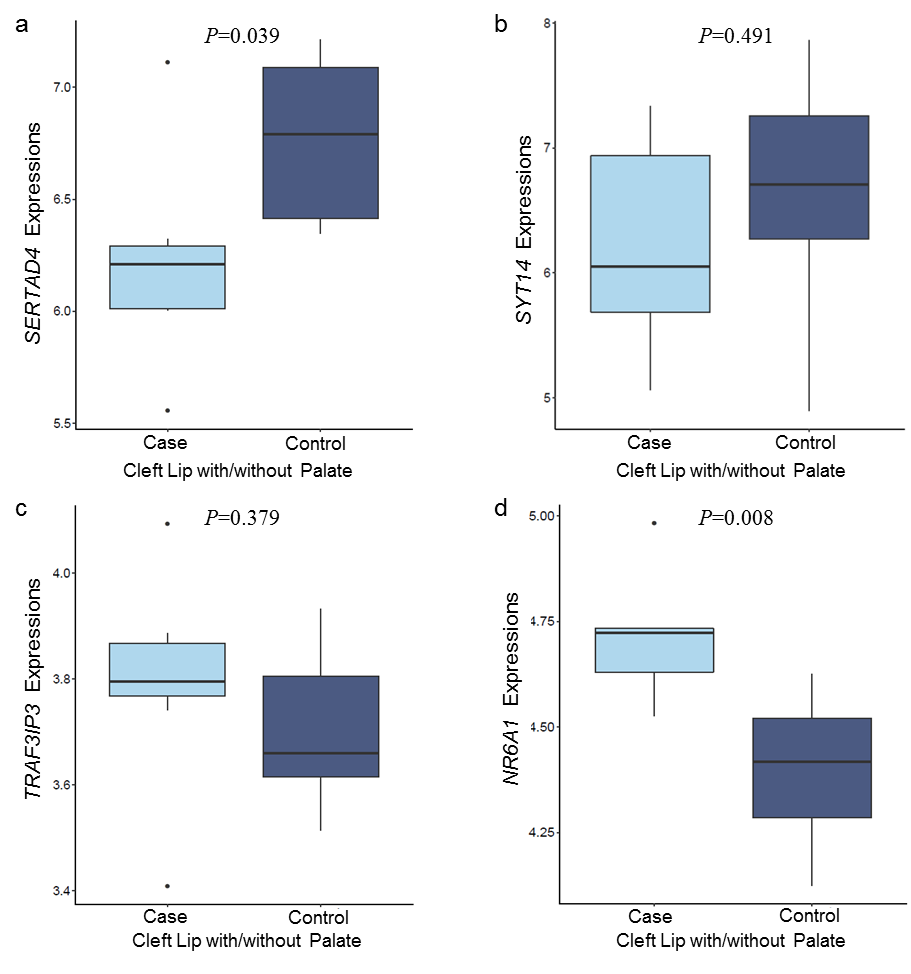


**Supplementary Figure 5**. Differential gene expression of dental pulp stem cells in NSCL/P patients and normal controls

**
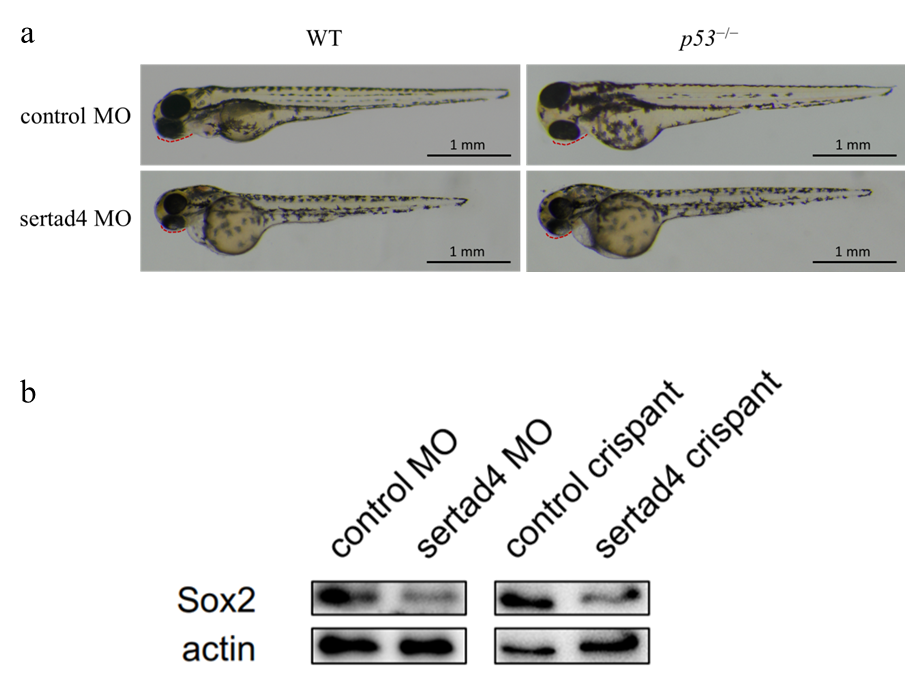
**

**Supplementary Figure 6**. (a) Comparison of the morphology of setrad4 MO injected wild-type (WT) and *p53*^-/-^ larvae at 96 hpf, (b) Sox2 protein expression levels in sertad4 MO or crispant zebrafish embryos at 96 hpf
