# Supplementary material for: Identification of novel susceptibility loci for non‐syndromic cleft lip with or without cleft palate
Source: J Cell Mol Med. 2020 Oct 27;24(23):13669–78. doi: 10.1111/jcmm.15878 (PMC7754035; doi:10.1111/jcmm.15878)
Supplement: Supplementary file 2 — Table S1‐S4 [file JCMM-24-13669-s002.docx]

| **Supplementary Table 1.** The primers and probes used in TaqMan genotyping assay | | |
| --- | --- | --- |
| SNP_ID | Primers | Probes |
| rs11119445 | F: CCCCTCTCCTGACACTAGTTATTGA | FAM-TTTACTCTACATGCATTCT (G allele) |
|  | R: GGCATTAGCCTTGACTTGGTTT | HEX-TTTACTCTACATACATTCTAAA (A allele) |
| rs227227 | F: CAACATTTGGCTGGTGTTTAGG | FAM-CATATTGAATGAGACAGTAT (G allele) |
|  | R: CGGTGTTGAATATGATTCCAGTGT | HEX-CCATATTGAATGAAAC (A allele) |
| rs12561877 | F: TTTCTGGACAGTTTCTTACCTTAGGAT | FAM-ATTCTGAGTGTATTCTAC (T allele) |
|  | R: CCTGCTTGCAGTTGTGAGAATAA | HEX-TTCTGAGTGCATTCTA (C allele) |
| rs643118 | F: CAGCATCTCCTATTGAGCATAACAG | FAM-CCCTGATGGAGTACC (G allele) |
|  | R: GTGTCCACTATGTTCCATGGTAATG | HEX-TAAGAAGCCCTGATAGAGTA (A allele) |
| rs2095293 | F: GATGCCAAAATCTACAGATGTTCAA | FAM-TGGATGCGATATAAGTGA (G allele) |
|  | R: AACAACCTCCCCCAACAAAAA | HEX-ATGCGATATAAATGATGTAA (A allele) |
| rs1925518 | F: GGGACTCAATAAATGGCTGTTGT | FAM-AGGATATTTAAGATACTTCAGTG (C allele) |
|  | R: CAGGGAAGGGTGTGCTAGGA | HEX-ATTTAAGATAATTCAGTGGTCAC (A allele) |

| **Supplementary Table 2.** The primary GWAS meta-analysis results for variants selected for replication | | | | | | | | | | |
| --- | --- | --- | --- | --- | --- | --- | --- | --- | --- | --- |
| CHR | BP | SNP | A1 | *P*_nanjing_ | *P*_huaxi_ | Meta-analysis | | | | |
|  |  |  |  |  |  | *P* | *P*(R) | OR | OR(R) | Q |
| 1 | 3003316 | rs2742676 | A | 1.94E-02 | 7.48E-03 | 3.90E-04 | 3.90E-04 | 1.31 | 1.31 | 0.89 |
| 1 | 4384859 | rs351609 | C | 2.57E-02 | 6.11E-03 | 4.08E-04 | 4.08E-04 | 1.33 | 1.33 | 0.96 |
| 1 | 5364634 | rs9439519 | C | 9.65E-03 | 2.21E-02 | 6.59E-04 | 6.59E-04 | 1.32 | 1.32 | 0.56 |
| 1 | 7713021 | rs9434879 | A | 1.84E-03 | 1.55E-02 | 1.21E-04 | 1.21E-04 | 0.72 | 0.72 | 0.37 |
| 1 | 10895024 | rs58177718 | A | 8.15E-03 | 3.04E-02 | 8.68E-04 | 8.68E-04 | 0.75 | 0.75 | 0.44 |
| 1 | 11496333 | rs12036240 | A | 1.95E-02 | 1.57E-02 | 8.30E-04 | 8.30E-04 | 0.78 | 0.78 | 0.72 |
| 1 | 11617025 | rs2817606 | T | 1.16E-02 | 1.67E-02 | 5.77E-04 | 5.77E-04 | 0.78 | 0.78 | 0.61 |
| 1 | 18797703 | rs2992736 | A | 3.43E-02 | 6.51E-03 | 5.72E-04 | 5.72E-04 | 0.75 | 0.75 | 0.92 |
| 1 | 22439520 | rs4654783 | C | 4.65E-03 | 3.80E-02 | 7.38E-04 | 7.38E-04 | 1.31 | 1.31 | 0.33 |
| 1 | 23015924 | rs75742397 | A | 6.28E-04 | 2.67E-02 | 1.08E-04 | 2.04E-03 | 1.86 | 1.89 | 0.20 |
| 1 | 31045509 | rs3104435 | A | 8.39E-03 | 1.79E-02 | 4.79E-04 | 4.79E-04 | 1.36 | 1.36 | 0.55 |
| 1 | 36795430 | rs4653178 | C | 2.65E-02 | 4.07E-04 | 3.18E-05 | 3.18E-05 | 1.44 | 1.44 | 0.72 |
| 1 | 36807452 | rs3795499 | A | 4.43E-03 | 2.93E-02 | 5.59E-04 | 5.59E-04 | 1.42 | 1.42 | 0.33 |
| 1 | 38577967 | rs528293 | A | 4.75E-03 | 3.23E-02 | 6.05E-04 | 6.05E-04 | 1.30 | 1.30 | 0.38 |
| 1 | 40513710 | rs3131683 | C | 4.15E-02 | 3.13E-03 | 3.43E-04 | 3.43E-04 | 1.30 | 1.30 | 0.81 |
| 1 | 53590423 | rs3766792 | T | 1.96E-02 | 8.16E-03 | 5.23E-04 | 5.23E-04 | 0.71 | 0.71 | 0.52 |
| 1 | 69926258 | rs1926269 | A | 2.21E-03 | 3.98E-02 | 4.84E-04 | 3.08E-03 | 0.77 | 0.77 | 0.23 |
| 1 | 73919673 | rs11210252 | A | 2.24E-02 | 6.70E-03 | 3.91E-04 | 3.91E-04 | 1.48 | 1.48 | 0.99 |
| 1 | 74444170 | rs11210386 | T | 1.33E-02 | 1.58E-02 | 6.56E-04 | 6.56E-04 | 0.68 | 0.68 | 0.56 |
| 1 | 86917944 | rs4656100 | T | 4.65E-04 | 2.37E-02 | 8.46E-05 | 3.74E-03 | 1.34 | 1.35 | 0.17 |
| 1 | 97664561 | rs75637747 | T | 1.59E-03 | 2.88E-02 | 2.25E-04 | 5.65E-04 | 0.76 | 0.76 | 0.28 |
| 1 | 99728806 | rs78826666 | G | 1.42E-02 | 1.67E-03 | 6.82E-05 | 6.82E-05 | 1.62 | 1.62 | 0.86 |
| 1 | 101072925 | rs17123012 | T | 1.30E-02 | 8.87E-03 | 3.76E-04 | 3.76E-04 | 1.71 | 1.71 | 0.55 |
| 1 | 157390112 | rs12745427 | C | 1.41E-03 | 2.02E-02 | 1.54E-04 | 7.04E-04 | 0.73 | 0.73 | 0.26 |
| 1 | 157453883 | rs6427381 | A | 1.26E-04 | 2.43E-02 | 3.58E-05 | 9.28E-03 | 1.36 | 1.39 | 0.10 |
| 1 | 172939896 | rs11579340 | A | 1.41E-03 | 2.32E-02 | 2.54E-04 | 8.17E-03 | 0.60 | 0.58 | 0.16 |
| 1 | 179564144 | rs10753200 | G | 3.88E-02 | 1.67E-03 | 1.79E-04 | 1.79E-04 | 0.73 | 0.73 | 0.75 |
| 1 | 179670125 | rs10798711 | T | 1.52E-02 | 1.03E-02 | 4.36E-04 | 4.36E-04 | 0.68 | 0.68 | 0.73 |
| 1 | 182577385 | rs55934782 | G | 8.01E-03 | 2.55E-02 | 6.73E-04 | 6.73E-04 | 0.77 | 0.77 | 0.50 |
| 1 | 193743268 | rs6428191 | T | 2.99E-02 | 7.97E-03 | 6.15E-04 | 6.15E-04 | 0.67 | 0.67 | 0.89 |
| 1 | 198097178 | rs16842420 | G | 1.26E-02 | 2.17E-02 | 8.77E-04 | 8.77E-04 | 1.35 | 1.35 | 0.52 |
| 1 | 208144550 | rs74688190 | A | 1.47E-03 | 3.78E-02 | 2.97E-04 | 1.64E-03 | 1.79 | 1.81 | 0.25 |
| 1 | 209885509 | rs4844488 | G | 5.13E-03 | 7.55E-03 | 1.33E-04 | 1.33E-04 | 0.72 | 0.72 | 0.54 |
| 1 | 209934841 | rs643118 | T | 1.17E-03 | 4.09E-02 | 3.00E-04 | 4.29E-03 | 1.36 | 1.37 | 0.20 |
| 1 | 209947885 | rs4140636 | C | 3.59E-05 | 1.39E-02 | 1.13E-05 | 1.77E-02 | 0.70 | 0.67 | 0.05 |
| 1 | 209977111 | rs861020 | A | 1.13E-03 | 3.46E-03 | 1.62E-05 | 1.62E-05 | 1.46 | 1.46 | 0.47 |
| 1 | 210027026 | rs6689839 | G | 6.41E-03 | 1.81E-02 | 3.94E-04 | 3.94E-04 | 1.34 | 1.34 | 0.51 |
| 1 | 210195000 | rs227227 | C | 8.16E-05 | 9.27E-03 | 1.03E-05 | 6.52E-03 | 1.39 | 1.42 | 0.09 |
| 1 | 210215414 | rs12561877 | T | 5.21E-04 | 2.72E-02 | 1.46E-04 | 1.30E-02 | 0.72 | 0.70 | 0.11 |
| 1 | 210355442 | rs7543674 | T | 1.99E-04 | 4.71E-02 | 1.19E-04 | 1.92E-02 | 1.41 | 1.45 | 0.09 |
| 1 | 210374337 | rs2485888 | A | 1.20E-03 | 3.09E-02 | 2.53E-04 | 5.07E-03 | 1.32 | 1.34 | 0.18 |
| 1 | 210397396 | rs11119445 | A | 1.08E-04 | 1.34E-02 | 1.99E-05 | 9.48E-03 | 0.73 | 0.71 | 0.09 |
| 1 | 216673326 | rs17659699 | G | 1.04E-03 | 9.42E-03 | 4.56E-05 | 4.56E-05 | 0.71 | 0.71 | 0.35 |
| 1 | 220662325 | rs12568419 | A | 1.19E-02 | 6.72E-03 | 2.25E-04 | 2.25E-04 | 1.32 | 1.32 | 0.82 |
| 1 | 241772881 | rs3765813 | T | 2.31E-02 | 1.21E-02 | 7.33E-04 | 7.33E-04 | 0.77 | 0.77 | 0.82 |
| 1 | 241773027 | rs3765814 | T | 8.47E-03 | 1.57E-03 | 3.89E-05 | 3.89E-05 | 1.40 | 1.40 | 0.91 |
| 2 | 16716042 | rs10856790 | C | 1.62E-03 | 4.65E-02 | 4.34E-04 | 3.76E-03 | 0.74 | 0.74 | 0.22 |
| 2 | 31627947 | rs206800 | G | 6.97E-03 | 6.81E-03 | 1.73E-04 | 1.73E-04 | 0.61 | 0.61 | 0.48 |
| 2 | 32018541 | rs1383023 | A | 4.45E-04 | 2.38E-02 | 1.10E-04 | 1.12E-02 | 0.59 | 0.56 | 0.12 |
| 2 | 35140010 | rs11887091 | A | 3.41E-02 | 1.02E-03 | 1.10E-04 | 1.10E-04 | 1.47 | 1.47 | 0.58 |
| 2 | 41275572 | rs10490373 | G | 1.56E-04 | 4.11E-02 | 7.30E-05 | 1.33E-02 | 1.35 | 1.37 | 0.10 |
| 2 | 44311580 | rs12614353 | C | 1.63E-02 | 6.33E-03 | 2.78E-04 | 2.78E-04 | 1.78 | 1.78 | 0.93 |
| 2 | 46537056 | rs4952818 | T | 2.70E-03 | 1.39E-02 | 1.61E-04 | 1.61E-04 | 0.67 | 0.67 | 0.37 |
| 2 | 46651624 | rs4953372 | C | 1.23E-02 | 2.89E-03 | 1.07E-04 | 1.07E-04 | 1.78 | 1.78 | 0.71 |
| 2 | 49222572 | rs6746533 | T | 4.88E-02 | 5.82E-03 | 7.03E-04 | 7.03E-04 | 0.78 | 0.78 | 0.90 |
| 2 | 50193483 | rs17039581 | C | 3.46E-03 | 2.63E-02 | 3.68E-04 | 3.68E-04 | 1.36 | 1.36 | 0.37 |
| 2 | 52614168 | rs10173797 | G | 4.49E-02 | 3.75E-03 | 4.41E-04 | 4.41E-04 | 1.30 | 1.30 | 0.77 |
| 2 | 54073171 | rs810909 | C | 6.84E-03 | 1.20E-02 | 2.62E-04 | 2.62E-04 | 1.30 | 1.30 | 0.58 |
| 2 | 59282442 | rs991965 | T | 5.42E-04 | 5.82E-03 | 1.64E-05 | 1.64E-05 | 1.36 | 1.36 | 0.32 |
| 2 | 81208973 | rs77153942 | A | 5.56E-03 | 3.67E-02 | 7.34E-04 | 7.34E-04 | 1.57 | 1.57 | 0.42 |
| 2 | 96422939 | rs1813381 | C | 6.43E-04 | 3.14E-02 | 1.66E-04 | 7.52E-03 | 0.75 | 0.73 | 0.15 |
| 2 | 99947225 | rs57169028 | T | 1.10E-02 | 6.72E-03 | 2.04E-04 | 2.04E-04 | 1.53 | 1.53 | 0.95 |
| 2 | 132561730 | rs4270398 | T | 2.54E-02 | 5.35E-03 | 3.56E-04 | 3.56E-04 | 0.74 | 0.74 | 1.00 |
| 2 | 152355258 | rs10497082 | C | 3.17E-02 | 3.86E-03 | 3.20E-04 | 3.20E-04 | 0.74 | 0.74 | 0.93 |
| 2 | 154312942 | rs74445175 | A | 4.15E-03 | 4.22E-02 | 5.79E-04 | 5.79E-04 | 1.58 | 1.58 | 0.49 |
| 2 | 168021121 | rs11893377 | T | 7.28E-04 | 3.47E-02 | 1.59E-04 | 2.58E-03 | 0.71 | 0.70 | 0.20 |
| 2 | 171387405 | rs4668271 | C | 5.19E-03 | 4.52E-02 | 8.49E-04 | 8.49E-04 | 1.33 | 1.33 | 0.41 |
| 2 | 176697376 | rs3731789 | T | 2.12E-02 | 9.44E-03 | 5.55E-04 | 5.55E-04 | 0.69 | 0.69 | 0.72 |
| 2 | 190440809 | rs34206448 | C | 1.41E-02 | 6.52E-03 | 2.55E-04 | 2.55E-04 | 0.68 | 0.68 | 0.82 |
| 2 | 211499367 | rs12694205 | G | 6.57E-03 | 1.40E-02 | 2.90E-04 | 2.90E-04 | 0.66 | 0.66 | 0.59 |
| 2 | 220634509 | rs1035680 | T | 1.02E-02 | 4.87E-03 | 1.46E-04 | 1.46E-04 | 0.74 | 0.74 | 0.74 |
| 2 | 220783322 | rs4672960 | T | 1.61E-03 | 4.89E-02 | 3.59E-04 | 6.03E-04 | 1.57 | 1.58 | 0.30 |
| 2 | 233941388 | rs11674347 | G | 1.24E-02 | 1.57E-02 | 6.13E-04 | 6.13E-04 | 0.75 | 0.75 | 0.56 |
| 2 | 235588230 | rs2053476 | A | 3.30E-02 | 2.63E-03 | 2.31E-04 | 2.31E-04 | 0.74 | 0.74 | 0.84 |
| 2 | 237045226 | rs1317659 | C | 6.09E-03 | 1.11E-02 | 2.32E-04 | 2.32E-04 | 1.31 | 1.31 | 0.52 |
| 3 | 29376106 | rs9864192 | A | 1.22E-02 | 2.49E-02 | 8.68E-04 | 8.68E-04 | 1.32 | 1.32 | 0.64 |
| 3 | 42474546 | rs77756479 | T | 4.42E-02 | 5.47E-03 | 6.07E-04 | 6.07E-04 | 1.51 | 1.51 | 0.94 |
| 3 | 57177195 | rs6801574 | C | 5.98E-03 | 1.30E-02 | 2.67E-04 | 2.67E-04 | 0.76 | 0.76 | 0.51 |
| 3 | 59649558 | rs6808992 | G | 4.63E-02 | 5.44E-03 | 6.36E-04 | 6.36E-04 | 1.30 | 1.30 | 0.88 |
| 3 | 60369522 | rs9842521 | A | 2.80E-02 | 8.81E-03 | 6.32E-04 | 6.32E-04 | 0.78 | 0.78 | 0.91 |
| 3 | 68475662 | rs13069343 | T | 3.83E-03 | 1.25E-02 | 1.75E-04 | 1.75E-04 | 1.33 | 1.33 | 0.47 |
| 3 | 73157367 | rs301538 | C | 7.22E-03 | 2.63E-02 | 6.43E-04 | 6.43E-04 | 1.34 | 1.34 | 0.48 |
| 3 | 77582429 | rs6806214 | T | 5.41E-03 | 8.64E-03 | 1.40E-04 | 1.40E-04 | 1.44 | 1.44 | 0.72 |
| 3 | 77688744 | rs77670627 | G | 4.31E-03 | 9.60E-03 | 1.46E-04 | 1.46E-04 | 1.40 | 1.40 | 0.53 |
| 3 | 84782923 | rs74564976 | A | 1.50E-03 | 8.25E-03 | 4.39E-05 | 4.39E-05 | 1.87 | 1.87 | 0.55 |
| 3 | 101104682 | rs4553955 | C | 1.69E-02 | 1.32E-02 | 6.05E-04 | 6.05E-04 | 1.39 | 1.39 | 0.74 |
| 3 | 103173411 | rs77446172 | T | 3.35E-02 | 7.69E-04 | 7.54E-05 | 7.54E-05 | 1.39 | 1.39 | 0.67 |
| 3 | 107307266 | rs1282401 | G | 3.05E-02 | 1.57E-04 | 1.57E-05 | 1.57E-05 | 0.58 | 0.58 | 0.57 |
| 3 | 133174449 | rs9865003 | T | 1.12E-02 | 2.01E-02 | 6.57E-04 | 6.57E-04 | 0.69 | 0.69 | 0.63 |
| 3 | 143638244 | rs116870358 | C | 3.82E-03 | 2.84E-02 | 5.39E-04 | 1.67E-03 | 1.50 | 1.51 | 0.27 |
| 3 | 149058681 | rs73168817 | A | 1.71E-02 | 2.05E-03 | 9.78E-05 | 9.78E-05 | 0.53 | 0.53 | 0.92 |
| 3 | 161170522 | rs12636206 | C | 3.84E-02 | 3.23E-03 | 3.27E-04 | 3.27E-04 | 0.72 | 0.72 | 0.82 |
| 3 | 164376301 | rs59570876 | C | 1.10E-02 | 2.50E-02 | 8.38E-04 | 8.38E-04 | 1.30 | 1.30 | 0.56 |
| 3 | 164428203 | rs12634812 | T | 1.57E-02 | 3.06E-03 | 1.33E-04 | 1.33E-04 | 1.48 | 1.48 | 0.92 |
| 3 | 164470226 | rs12485526 | C | 2.25E-02 | 1.33E-02 | 7.94E-04 | 7.94E-04 | 1.28 | 1.28 | 0.80 |
| 3 | 171153434 | rs1491666 | C | 2.06E-03 | 4.35E-02 | 4.77E-04 | 2.54E-03 | 0.75 | 0.74 | 0.24 |
| 3 | 176525744 | rs74710876 | T | 7.58E-03 | 7.25E-03 | 1.88E-04 | 1.88E-04 | 1.45 | 1.45 | 0.52 |
| 3 | 177936807 | rs6776221 | G | 1.26E-02 | 1.28E-02 | 4.23E-04 | 4.23E-04 | 1.66 | 1.66 | 0.94 |
| 4 | 4852354 | rs884690 | G | 3.98E-02 | 7.04E-03 | 7.02E-04 | 7.02E-04 | 1.28 | 1.28 | 0.97 |
| 4 | 14044740 | rs16889667 | A | 1.35E-02 | 4.81E-03 | 1.81E-04 | 1.81E-04 | 0.73 | 0.73 | 0.84 |
| 4 | 17054262 | rs16894263 | A | 4.19E-02 | 3.66E-03 | 4.08E-04 | 4.08E-04 | 1.63 | 1.63 | 0.77 |
| 4 | 25144636 | rs3796794 | C | 2.22E-02 | 7.94E-03 | 4.64E-04 | 4.64E-04 | 1.51 | 1.51 | 0.88 |
| 4 | 27080119 | rs13139604 | T | 7.11E-03 | 1.62E-03 | 3.39E-05 | 3.39E-05 | 1.58 | 1.58 | 0.92 |
| 4 | 54176611 | rs17082579 | T | 1.12E-02 | 1.17E-02 | 3.71E-04 | 3.71E-04 | 0.68 | 0.68 | 0.72 |
| 4 | 58097310 | rs79121333 | T | 7.84E-03 | 1.93E-02 | 5.62E-04 | 5.62E-04 | 1.33 | 1.33 | 0.42 |
| 4 | 70112313 | rs10032551 | A | 3.28E-03 | 1.06E-02 | 1.48E-04 | 1.48E-04 | 1.30 | 1.30 | 0.38 |
| 4 | 72429371 | rs2290399 | G | 2.47E-02 | 5.09E-03 | 3.36E-04 | 3.36E-04 | 1.36 | 1.36 | 0.92 |
| 4 | 77509140 | rs4413433 | A | 1.40E-02 | 2.39E-02 | 9.92E-04 | 9.92E-04 | 1.32 | 1.32 | 0.59 |
| 4 | 77531982 | rs56408679 | C | 1.66E-02 | 9.49E-03 | 4.32E-04 | 4.32E-04 | 1.46 | 1.46 | 0.79 |
| 4 | 78947327 | rs7667122 | T | 1.73E-02 | 1.67E-02 | 7.91E-04 | 7.91E-04 | 1.28 | 1.28 | 0.69 |
| 4 | 85204825 | rs12331554 | G | 2.29E-02 | 1.56E-02 | 9.14E-04 | 9.14E-04 | 1.56 | 1.56 | 0.84 |
| 4 | 112281230 | rs67862869 | A | 2.09E-03 | 1.79E-02 | 1.86E-04 | 4.18E-04 | 0.71 | 0.71 | 0.29 |
| 4 | 120922444 | rs13130657 | C | 3.62E-02 | 5.18E-03 | 4.76E-04 | 4.76E-04 | 0.68 | 0.68 | 0.95 |
| 4 | 139525415 | rs10032519 | C | 1.51E-02 | 3.00E-03 | 1.24E-04 | 1.24E-04 | 1.33 | 1.33 | 0.95 |
| 4 | 139759878 | rs61388673 | C | 2.06E-02 | 5.87E-03 | 3.21E-04 | 3.21E-04 | 1.47 | 1.47 | 0.99 |
| 4 | 145612551 | rs63121460 | A | 3.68E-02 | 2.86E-03 | 2.82E-04 | 2.82E-04 | 1.31 | 1.31 | 0.81 |
| 4 | 152679542 | rs17027846 | G | 4.79E-03 | 3.06E-03 | 4.42E-05 | 4.42E-05 | 0.53 | 0.53 | 0.83 |
| 4 | 157705551 | rs7672622 | G | 1.92E-02 | 7.88E-03 | 4.09E-04 | 4.09E-04 | 0.73 | 0.73 | 0.82 |
| 4 | 164038379 | rs10019053 | C | 1.84E-02 | 1.71E-02 | 8.69E-04 | 8.69E-04 | 0.68 | 0.68 | 0.70 |
| 4 | 181300520 | rs10520447 | A | 2.06E-02 | 9.99E-03 | 5.46E-04 | 5.46E-04 | 1.40 | 1.40 | 0.82 |
| 4 | 182919753 | rs11946900 | A | 1.59E-02 | 6.43E-03 | 2.82E-04 | 2.82E-04 | 1.34 | 1.34 | 0.81 |
| 5 | 6082486 | rs17770545 | T | 2.87E-02 | 6.65E-03 | 4.89E-04 | 4.89E-04 | 0.71 | 0.71 | 0.99 |
| 5 | 6165158 | rs11134142 | A | 9.90E-03 | 2.03E-02 | 5.96E-04 | 5.96E-04 | 1.30 | 1.30 | 0.60 |
| 5 | 10247010 | rs2548554 | T | 8.20E-03 | 1.53E-02 | 4.28E-04 | 4.28E-04 | 1.45 | 1.45 | 0.49 |
| 5 | 23623207 | rs7705488 | T | 2.02E-02 | 3.54E-03 | 1.93E-04 | 1.93E-04 | 0.74 | 0.74 | 0.95 |
| 5 | 80459446 | rs7703816 | T | 4.74E-02 | 4.54E-03 | 5.59E-04 | 5.59E-04 | 1.58 | 1.58 | 0.77 |
| 5 | 82834299 | rs188703 | A | 2.00E-02 | 5.85E-03 | 3.09E-04 | 3.09E-04 | 1.33 | 1.33 | 0.94 |
| 5 | 82871546 | rs309581 | C | 1.83E-03 | 1.02E-02 | 8.30E-05 | 8.30E-05 | 1.34 | 1.34 | 0.37 |
| 5 | 92171562 | rs12188937 | C | 1.20E-02 | 2.14E-02 | 7.01E-04 | 7.01E-04 | 1.38 | 1.38 | 0.74 |
| 5 | 115298977 | rs1445708 | T | 2.72E-03 | 2.96E-02 | 3.48E-04 | 3.48E-04 | 0.77 | 0.77 | 0.33 |
| 5 | 116693237 | rs11738865 | A | 3.25E-03 | 3.17E-02 | 4.50E-04 | 4.50E-04 | 1.68 | 1.68 | 0.32 |
| 5 | 127876229 | rs35079125 | A | 1.45E-03 | 2.81E-03 | 1.51E-05 | 1.51E-05 | 1.89 | 1.89 | 0.55 |
| 5 | 127898518 | rs10055140 | G | 6.77E-03 | 4.11E-03 | 8.93E-05 | 8.93E-05 | 1.46 | 1.46 | 0.66 |
| 5 | 145116924 | rs74679696 | T | 2.51E-03 | 2.47E-02 | 2.95E-04 | 4.86E-04 | 0.62 | 0.61 | 0.30 |
| 5 | 147835912 | rs2037997 | A | 6.05E-03 | 5.53E-03 | 1.02E-04 | 1.02E-04 | 1.33 | 1.33 | 0.71 |
| 5 | 148776470 | rs353266 | T | 2.14E-02 | 9.53E-03 | 5.49E-04 | 5.49E-04 | 0.71 | 0.71 | 0.78 |
| 5 | 149854482 | rs73272621 | G | 4.94E-02 | 1.84E-03 | 2.55E-04 | 2.55E-04 | 1.32 | 1.32 | 0.67 |
| 5 | 169328238 | rs6883554 | C | 2.65E-02 | 6.61E-03 | 4.55E-04 | 4.55E-04 | 0.72 | 0.72 | 0.99 |
| 5 | 174930452 | rs11134927 | A | 9.91E-04 | 2.90E-02 | 1.99E-04 | 4.39E-03 | 1.32 | 1.33 | 0.18 |
| 5 | 176378574 | rs365132 | G | 3.42E-02 | 9.16E-03 | 7.85E-04 | 7.85E-04 | 0.78 | 0.78 | 0.99 |
| 6 | 1929894 | rs12194687 | G | 1.04E-02 | 1.44E-02 | 4.81E-04 | 4.81E-04 | 1.38 | 1.38 | 0.55 |
| 6 | 6808208 | rs4960248 | T | 1.34E-03 | 3.90E-02 | 3.19E-04 | 3.85E-03 | 0.77 | 0.76 | 0.21 |
| 6 | 8645733 | rs10484744 | A | 2.28E-02 | 1.74E-02 | 9.92E-04 | 9.92E-04 | 1.61 | 1.61 | 0.91 |
| 6 | 14664991 | rs7761103 | T | 6.68E-03 | 4.02E-02 | 9.39E-04 | 9.39E-04 | 1.33 | 1.33 | 0.44 |
| 6 | 15927601 | rs58093807 | C | 4.83E-02 | 3.37E-03 | 4.29E-04 | 4.29E-04 | 0.77 | 0.77 | 0.77 |
| 6 | 23954438 | rs9348623 | G | 3.89E-02 | 5.87E-03 | 5.71E-04 | 5.71E-04 | 1.47 | 1.47 | 0.96 |
| 6 | 23972434 | rs36072781 | A | 2.14E-02 | 3.95E-03 | 2.26E-04 | 2.26E-04 | 1.57 | 1.57 | 0.90 |
| 6 | 25414402 | rs72831239 | C | 2.46E-03 | 1.55E-02 | 1.61E-04 | 1.61E-04 | 0.72 | 0.72 | 0.38 |
| 6 | 31262869 | rs9357123 | A | 1.10E-04 | 1.92E-02 | 3.89E-05 | 2.05E-02 | 0.70 | 0.67 | 0.06 |
| 6 | 31318164 | rs4394274 | A | 4.89E-03 | 1.96E-02 | 3.53E-04 | 3.53E-04 | 1.30 | 1.30 | 0.45 |
| 6 | 31320930 | rs77616974 | G | 1.64E-02 | 1.58E-02 | 6.72E-04 | 6.72E-04 | 1.52 | 1.52 | 0.87 |
| 6 | 32396615 | rs3135342 | T | 2.21E-02 | 5.69E-03 | 3.33E-04 | 3.33E-04 | 1.50 | 1.50 | 0.99 |
| 6 | 32609312 | rs1129808 | A | 1.27E-02 | 9.92E-03 | 3.76E-04 | 3.76E-04 | 1.31 | 1.31 | 0.64 |
| 6 | 89916641 | rs7758893 | T | 4.03E-02 | 7.68E-03 | 7.70E-04 | 7.70E-04 | 0.78 | 0.78 | 0.96 |
| 6 | 106126253 | rs845861 | T | 2.95E-02 | 8.16E-03 | 6.13E-04 | 6.13E-04 | 0.78 | 0.78 | 0.96 |
| 6 | 109106052 | rs79642703 | A | 3.25E-03 | 6.57E-03 | 7.98E-05 | 7.98E-05 | 0.67 | 0.67 | 0.49 |
| 6 | 123620680 | rs9490721 | G | 2.34E-02 | 8.08E-03 | 4.96E-04 | 4.96E-04 | 1.31 | 1.31 | 0.90 |
| 6 | 127726196 | rs6569487 | A | 3.27E-02 | 3.00E-03 | 2.58E-04 | 2.58E-04 | 1.31 | 1.31 | 0.90 |
| 6 | 127805089 | rs1512449 | A | 2.28E-03 | 1.44E-02 | 1.30E-04 | 1.30E-04 | 1.36 | 1.36 | 0.42 |
| 6 | 132172368 | rs1044498 | C | 4.54E-02 | 5.80E-03 | 6.63E-04 | 6.63E-04 | 1.47 | 1.47 | 0.85 |
| 6 | 145400233 | rs650555 | C | 4.97E-03 | 1.55E-02 | 2.89E-04 | 2.89E-04 | 1.32 | 1.32 | 0.44 |
| 6 | 151427385 | rs6934983 | G | 9.30E-03 | 1.64E-02 | 4.58E-04 | 4.58E-04 | 1.61 | 1.61 | 0.62 |
| 6 | 162086942 | rs7771341 | C | 7.14E-03 | 3.38E-02 | 8.69E-04 | 8.69E-04 | 0.75 | 0.75 | 0.42 |
| 6 | 162906921 | rs1893113 | T | 3.28E-02 | 2.06E-04 | 2.33E-05 | 2.33E-05 | 0.73 | 0.73 | 0.51 |
| 6 | 165179436 | rs9459195 | A | 2.04E-03 | 4.13E-02 | 4.48E-04 | 2.30E-03 | 1.30 | 1.30 | 0.25 |
| 6 | 170392739 | rs4710845 | A | 5.18E-04 | 1.21E-02 | 4.19E-05 | 9.51E-04 | 1.37 | 1.38 | 0.21 |
| 7 | 10976311 | rs12537541 | G | 1.93E-03 | 5.83E-03 | 4.13E-05 | 4.13E-05 | 1.79 | 1.79 | 0.52 |
| 7 | 15606338 | rs10239845 | G | 3.43E-02 | 1.06E-02 | 9.17E-04 | 9.17E-04 | 0.78 | 0.78 | 0.89 |
| 7 | 17309279 | rs12670403 | A | 1.51E-02 | 1.39E-02 | 5.90E-04 | 5.90E-04 | 0.78 | 0.78 | 0.70 |
| 7 | 22045685 | rs1730886 | C | 1.19E-02 | 1.51E-02 | 5.38E-04 | 5.38E-04 | 1.29 | 1.29 | 0.62 |
| 7 | 22534805 | rs6972788 | C | 2.02E-02 | 1.24E-02 | 6.58E-04 | 6.58E-04 | 0.78 | 0.78 | 0.83 |
| 7 | 26590857 | rs1859601 | T | 2.91E-02 | 1.26E-02 | 9.23E-04 | 9.23E-04 | 0.73 | 0.73 | 0.88 |
| 7 | 26626078 | rs6973914 | T | 1.78E-02 | 4.38E-03 | 2.12E-04 | 2.12E-04 | 0.71 | 0.71 | 0.89 |
| 7 | 37908837 | rs3807172 | T | 1.18E-02 | 1.21E-02 | 4.25E-04 | 4.25E-04 | 0.62 | 0.62 | 0.64 |
| 7 | 42424267 | rs10260653 | G | 1.59E-03 | 1.00E-02 | 6.92E-05 | 6.92E-05 | 1.35 | 1.35 | 0.38 |
| 7 | 66521879 | rs148150768 | T | 1.29E-02 | 2.26E-02 | 7.97E-04 | 7.97E-04 | 1.40 | 1.40 | 0.70 |
| 7 | 66671768 | rs4718479 | A | 1.21E-02 | 4.71E-03 | 1.59E-04 | 1.59E-04 | 1.34 | 1.34 | 0.85 |
| 7 | 66773439 | rs17146158 | A | 1.17E-03 | 2.55E-03 | 1.10E-05 | 1.10E-05 | 1.58 | 1.58 | 0.57 |
| 7 | 66774157 | rs2041575 | T | 7.14E-03 | 2.92E-02 | 7.28E-04 | 7.28E-04 | 0.76 | 0.76 | 0.45 |
| 7 | 66811313 | rs7804537 | A | 2.13E-03 | 7.79E-03 | 6.24E-05 | 6.24E-05 | 1.41 | 1.41 | 0.48 |
| 7 | 70718834 | rs17593949 | A | 4.95E-02 | 3.42E-03 | 4.38E-04 | 4.38E-04 | 1.46 | 1.46 | 0.81 |
| 7 | 78106455 | rs11764471 | G | 5.35E-03 | 3.33E-02 | 6.24E-04 | 6.24E-04 | 1.36 | 1.36 | 0.44 |
| 7 | 87768174 | rs10268574 | G | 2.06E-02 | 8.06E-03 | 4.36E-04 | 4.36E-04 | 1.30 | 1.30 | 0.88 |
| 7 | 103732511 | rs11772175 | A | 3.60E-02 | 5.57E-03 | 5.12E-04 | 5.12E-04 | 1.41 | 1.41 | 0.91 |
| 7 | 103771799 | rs194845 | G | 4.64E-03 | 2.32E-02 | 4.02E-04 | 4.02E-04 | 0.77 | 0.77 | 0.42 |
| 7 | 105700868 | rs10487831 | T | 2.49E-02 | 5.50E-03 | 3.59E-04 | 3.59E-04 | 0.70 | 0.70 | 0.96 |
| 7 | 127077326 | rs10250993 | C | 7.47E-03 | 1.00E-02 | 2.18E-04 | 2.18E-04 | 1.68 | 1.68 | 0.74 |
| 7 | 143071480 | rs10226669 | T | 3.07E-02 | 3.78E-03 | 3.06E-04 | 3.06E-04 | 0.70 | 0.70 | 0.87 |
| 7 | 153461606 | rs6962199 | T | 1.08E-03 | 3.04E-02 | 2.07E-04 | 3.05E-03 | 1.34 | 1.36 | 0.20 |
| 7 | 155820045 | rs12671032 | T | 6.56E-03 | 2.42E-03 | 4.80E-05 | 4.80E-05 | 0.72 | 0.72 | 0.82 |
| 8 | 6679796 | rs9774066 | C | 4.73E-02 | 6.28E-03 | 7.47E-04 | 7.47E-04 | 1.60 | 1.60 | 0.83 |
| 8 | 13454638 | rs1481592 | C | 2.45E-02 | 7.98E-04 | 5.71E-05 | 5.71E-05 | 1.37 | 1.37 | 0.74 |
| 8 | 14748493 | rs2726607 | T | 1.91E-02 | 1.79E-02 | 9.41E-04 | 9.41E-04 | 0.73 | 0.73 | 0.69 |
| 8 | 17027279 | rs17687043 | A | 1.29E-02 | 2.31E-02 | 9.09E-04 | 9.09E-04 | 1.29 | 1.29 | 0.57 |
| 8 | 18572835 | rs7009615 | C | 4.48E-02 | 2.90E-03 | 3.36E-04 | 3.36E-04 | 0.77 | 0.77 | 0.86 |
| 8 | 26634589 | rs6557946 | A | 8.81E-03 | 1.93E-02 | 5.03E-04 | 5.03E-04 | 1.49 | 1.49 | 0.64 |
| 8 | 48492845 | rs2171854 | C | 2.18E-03 | 1.77E-02 | 1.66E-04 | 1.66E-04 | 1.48 | 1.48 | 0.36 |
| 8 | 70362479 | rs7011706 | T | 1.46E-02 | 1.36E-02 | 5.73E-04 | 5.73E-04 | 0.77 | 0.77 | 0.67 |
| 8 | 73916876 | rs10098931 | A | 4.30E-02 | 6.16E-03 | 6.66E-04 | 6.66E-04 | 1.33 | 1.33 | 0.92 |
| 8 | 85330924 | rs7010781 | G | 4.32E-02 | 5.61E-03 | 6.07E-04 | 6.07E-04 | 0.78 | 0.78 | 0.96 |
| 8 | 85360962 | rs711007 | G | 4.00E-02 | 9.18E-03 | 9.09E-04 | 9.09E-04 | 0.78 | 0.78 | 0.95 |
| 8 | 88965081 | rs2337161 | T | 4.77E-02 | 2.67E-03 | 3.37E-04 | 3.37E-04 | 0.76 | 0.76 | 0.76 |
| 8 | 99794459 | rs4255105 | A | 9.02E-03 | 2.12E-02 | 6.02E-04 | 6.02E-04 | 1.28 | 1.28 | 0.56 |
| 8 | 100194295 | rs35018432 | T | 3.87E-03 | 1.14E-03 | 1.38E-05 | 1.38E-05 | 1.38 | 1.38 | 0.80 |
| 8 | 100638489 | rs6985574 | T | 8.12E-03 | 6.73E-04 | 1.63E-05 | 1.63E-05 | 1.38 | 1.38 | 0.97 |
| 8 | 104194150 | rs7817115 | A | 2.84E-02 | 1.06E-03 | 8.73E-05 | 8.73E-05 | 1.36 | 1.36 | 0.72 |
| 8 | 129981358 | rs11997787 | T | 3.30E-02 | 5.15E-03 | 4.64E-04 | 4.64E-04 | 1.54 | 1.54 | 0.75 |
| 8 | 135419683 | rs7011450 | G | 6.12E-03 | 2.07E-02 | 4.31E-04 | 4.31E-04 | 0.76 | 0.76 | 0.49 |
| 8 | 144887772 | rs7844493 | G | 2.52E-03 | 7.51E-03 | 7.06E-05 | 7.06E-05 | 1.35 | 1.35 | 0.48 |
| 9 | 8704192 | rs1550002 | C | 6.93E-03 | 1.67E-02 | 3.99E-04 | 3.99E-04 | 1.30 | 1.30 | 0.49 |
| 9 | 23431323 | rs10122484 | A | 2.78E-02 | 8.57E-03 | 6.07E-04 | 6.07E-04 | 0.74 | 0.74 | 0.99 |
| 9 | 24247485 | rs17197195 | A | 3.19E-03 | 3.39E-02 | 5.00E-04 | 7.71E-04 | 1.35 | 1.36 | 0.30 |
| 9 | 24319964 | rs1576328 | C | 7.55E-03 | 3.44E-02 | 8.44E-04 | 8.44E-04 | 1.53 | 1.53 | 0.49 |
| 9 | 28120384 | rs1452333 | C | 1.56E-02 | 1.96E-02 | 8.84E-04 | 8.84E-04 | 1.49 | 1.49 | 0.62 |
| 9 | 30101681 | rs2800322 | G | 1.08E-02 | 2.17E-02 | 7.09E-04 | 7.09E-04 | 1.29 | 1.29 | 0.60 |
| 9 | 38496889 | rs4598351 | T | 3.90E-02 | 9.66E-03 | 9.30E-04 | 9.30E-04 | 0.60 | 0.60 | 0.99 |
| 9 | 83472648 | rs1547137 | T | 1.27E-02 | 1.14E-02 | 4.23E-04 | 4.23E-04 | 0.77 | 0.77 | 0.67 |
| 9 | 90450644 | rs10116815 | T | 6.77E-03 | 2.15E-02 | 4.68E-04 | 4.68E-04 | 1.31 | 1.31 | 0.53 |
| 9 | 92709135 | rs75788647 | A | 6.31E-03 | 3.68E-02 | 7.78E-04 | 7.78E-04 | 1.35 | 1.35 | 0.47 |
| 9 | 96902441 | rs55990139 | T | 1.52E-02 | 1.94E-02 | 8.35E-04 | 8.35E-04 | 1.29 | 1.29 | 0.64 |
| 9 | 102479796 | rs80347791 | A | 3.65E-02 | 7.65E-03 | 7.00E-04 | 7.00E-04 | 1.70 | 1.70 | 0.97 |
| 9 | 107240501 | rs10991273 | A | 1.80E-02 | 4.65E-03 | 2.31E-04 | 2.31E-04 | 1.37 | 1.37 | 0.82 |
| 9 | 108418255 | rs16924806 | C | 2.43E-02 | 9.03E-03 | 5.63E-04 | 5.63E-04 | 1.29 | 1.29 | 0.89 |
| 9 | 115797957 | rs62574388 | A | 4.58E-03 | 3.31E-02 | 6.20E-04 | 6.20E-04 | 0.60 | 0.60 | 0.35 |
| 9 | 127291112 | rs2095293 | T | 2.36E-02 | 1.21E-02 | 7.40E-04 | 7.40E-04 | 1.32 | 1.32 | 0.85 |
| 10 | 8089136 | rs4143094 | T | 2.25E-02 | 8.44E-03 | 4.89E-04 | 4.89E-04 | 1.64 | 1.64 | 0.98 |
| 10 | 9955897 | rs7075973 | G | 4.68E-03 | 1.91E-02 | 3.19E-04 | 3.19E-04 | 1.30 | 1.30 | 0.46 |
| 10 | 28605434 | rs7074005 | G | 6.83E-04 | 3.93E-02 | 2.14E-04 | 8.52E-03 | 0.76 | 0.75 | 0.15 |
| 10 | 50532588 | rs10857469 | G | 1.68E-02 | 1.58E-02 | 7.51E-04 | 7.51E-04 | 1.46 | 1.46 | 0.66 |
| 10 | 67067501 | rs10822486 | C | 4.02E-02 | 2.42E-03 | 2.62E-04 | 2.62E-04 | 1.31 | 1.31 | 0.79 |
| 10 | 77213547 | rs7912002 | A | 4.86E-03 | 1.23E-02 | 1.89E-04 | 1.89E-04 | 1.37 | 1.37 | 0.62 |
| 10 | 94899090 | rs7081913 | A | 3.13E-02 | 5.97E-03 | 4.88E-04 | 4.88E-04 | 1.70 | 1.70 | 0.84 |
| 10 | 111938114 | rs12416245 | G | 6.63E-03 | 1.15E-02 | 2.26E-04 | 2.26E-04 | 1.34 | 1.34 | 0.68 |
| 10 | 118611836 | rs76060179 | T | 5.58E-03 | 8.29E-03 | 1.64E-04 | 1.64E-04 | 0.63 | 0.63 | 0.51 |
| 10 | 118805099 | rs4123200 | G | 4.79E-02 | 4.94E-05 | 1.02E-05 | 1.02E-05 | 0.60 | 0.60 | 0.34 |
| 10 | 118861724 | rs11197899 | T | 1.98E-02 | 1.66E-04 | 1.05E-05 | 1.05E-05 | 1.38 | 1.38 | 0.66 |
| 10 | 123092807 | rs4752536 | A | 6.50E-03 | 1.09E-02 | 2.30E-04 | 2.30E-04 | 0.76 | 0.76 | 0.57 |
| 10 | 131539740 | rs11592693 | T | 4.95E-02 | 9.45E-04 | 1.42E-04 | 1.42E-04 | 0.64 | 0.64 | 0.58 |
| 11 | 27453752 | rs79811440 | C | 1.15E-02 | 1.97E-03 | 6.61E-05 | 6.61E-05 | 1.67 | 1.67 | 0.85 |
| 11 | 44302865 | rs74235680 | T | 1.10E-02 | 2.31E-02 | 7.15E-04 | 7.15E-04 | 1.61 | 1.61 | 0.68 |
| 11 | 44312648 | rs2863058 | G | 1.36E-02 | 8.40E-04 | 3.47E-05 | 3.47E-05 | 1.69 | 1.69 | 0.75 |
| 11 | 65912524 | rs17147371 | C | 2.75E-02 | 1.03E-02 | 7.38E-04 | 7.38E-04 | 0.74 | 0.74 | 0.81 |
| 11 | 76333308 | rs7113568 | T | 3.13E-04 | 4.75E-02 | 1.43E-04 | 1.21E-02 | 1.54 | 1.58 | 0.12 |
| 11 | 91885496 | rs978876 | C | 4.05E-04 | 3.00E-02 | 9.83E-05 | 5.40E-03 | 1.78 | 1.83 | 0.15 |
| 11 | 94671229 | rs7932014 | G | 7.68E-03 | 2.43E-02 | 5.90E-04 | 5.90E-04 | 0.77 | 0.77 | 0.54 |
| 11 | 97118867 | rs964646 | C | 2.26E-03 | 2.20E-02 | 2.58E-04 | 8.86E-04 | 0.66 | 0.66 | 0.27 |
| 11 | 98897790 | rs11218104 | A | 1.32E-02 | 2.64E-02 | 9.92E-04 | 9.92E-04 | 0.68 | 0.68 | 0.64 |
| 11 | 102150488 | rs2604532 | A | 1.99E-03 | 4.14E-02 | 4.29E-04 | 2.03E-03 | 1.32 | 1.33 | 0.25 |
| 11 | 134792477 | rs1478748 | C | 9.39E-04 | 2.18E-02 | 1.23E-04 | 1.37E-03 | 0.75 | 0.75 | 0.23 |
| 11 | 134821370 | rs11224103 | T | 1.09E-02 | 1.56E-02 | 5.16E-04 | 5.16E-04 | 1.31 | 1.31 | 0.61 |
| 11 | 134930689 | rs4540845 | A | 1.28E-02 | 2.33E-02 | 8.98E-04 | 8.98E-04 | 0.74 | 0.74 | 0.57 |
| 12 | 3233526 | rs74462168 | C | 1.63E-02 | 1.47E-02 | 6.72E-04 | 6.72E-04 | 1.54 | 1.54 | 0.71 |
| 12 | 21326672 | rs12320965 | C | 1.12E-02 | 5.78E-03 | 1.82E-04 | 1.82E-04 | 1.37 | 1.37 | 0.83 |
| 12 | 21404167 | rs9804727 | G | 5.50E-03 | 1.29E-02 | 2.21E-04 | 2.21E-04 | 1.36 | 1.36 | 0.62 |
| 12 | 23752395 | rs752684 | T | 1.02E-03 | 2.17E-02 | 1.27E-04 | 9.78E-04 | 0.74 | 0.74 | 0.24 |
| 12 | 23819364 | rs10771017 | A | 2.23E-02 | 5.00E-03 | 2.96E-04 | 2.96E-04 | 0.76 | 0.76 | 0.94 |
| 12 | 28744557 | rs10771436 | G | 3.01E-02 | 8.56E-03 | 6.57E-04 | 6.57E-04 | 1.29 | 1.29 | 0.97 |
| 12 | 28744598 | rs55902604 | C | 8.22E-03 | 2.66E-02 | 7.49E-04 | 7.49E-04 | 0.77 | 0.77 | 0.46 |
| 12 | 31169728 | rs2016273 | A | 2.32E-02 | 9.31E-03 | 5.93E-04 | 5.93E-04 | 1.51 | 1.51 | 0.73 |
| 12 | 38514872 | rs1969363 | G | 7.51E-03 | 3.78E-02 | 9.99E-04 | 9.99E-04 | 1.28 | 1.28 | 0.42 |
| 12 | 42384593 | rs78038704 | T | 1.19E-02 | 2.53E-02 | 8.73E-04 | 8.73E-04 | 1.46 | 1.46 | 0.63 |
| 12 | 46940981 | rs17097002 | A | 3.72E-03 | 2.89E-02 | 5.37E-04 | 1.63E-03 | 1.46 | 1.47 | 0.27 |
| 12 | 51681903 | rs7954976 | T | 5.20E-03 | 4.17E-02 | 8.45E-04 | 8.45E-04 | 1.65 | 1.65 | 0.37 |
| 12 | 55048199 | rs10876578 | G | 9.37E-03 | 1.56E-02 | 4.67E-04 | 4.67E-04 | 0.75 | 0.75 | 0.55 |
| 12 | 59279660 | rs11172796 | T | 1.36E-02 | 1.44E-02 | 5.36E-04 | 5.36E-04 | 1.46 | 1.46 | 0.75 |
| 12 | 63259895 | rs599969 | G | 3.04E-02 | 5.87E-03 | 4.60E-04 | 4.60E-04 | 0.78 | 0.78 | 0.99 |
| 12 | 63277029 | rs1695003 | A | 6.27E-03 | 3.64E-02 | 8.35E-04 | 8.35E-04 | 0.78 | 0.78 | 0.41 |
| 12 | 71439825 | rs1913201 | A | 4.30E-02 | 6.99E-03 | 7.48E-04 | 7.48E-04 | 0.77 | 0.77 | 0.91 |
| 12 | 75567231 | rs74870575 | C | 5.82E-03 | 1.85E-02 | 3.64E-04 | 3.64E-04 | 1.49 | 1.49 | 0.50 |
| 12 | 75944907 | rs4882631 | T | 4.93E-03 | 3.09E-02 | 5.63E-04 | 5.63E-04 | 0.78 | 0.78 | 0.41 |
| 12 | 95687486 | rs7309251 | G | 3.71E-03 | 2.53E-02 | 3.39E-04 | 3.39E-04 | 0.66 | 0.66 | 0.45 |
| 12 | 106401180 | rs78969953 | T | 3.85E-02 | 1.28E-03 | 1.61E-04 | 1.61E-04 | 1.50 | 1.50 | 0.51 |
| 12 | 109537564 | rs2541886 | T | 1.37E-02 | 2.15E-02 | 8.49E-04 | 8.49E-04 | 1.36 | 1.36 | 0.62 |
| 12 | 125141715 | rs1726254 | G | 1.28E-02 | 8.42E-04 | 3.14E-05 | 3.14E-05 | 0.74 | 0.74 | 0.89 |
| 12 | 130629493 | rs10773740 | T | 1.23E-03 | 3.50E-02 | 3.01E-04 | 6.42E-03 | 0.73 | 0.72 | 0.18 |
| 13 | 23760912 | rs55669067 | G | 1.82E-02 | 7.23E-03 | 3.56E-04 | 3.56E-04 | 0.66 | 0.66 | 0.83 |
| 13 | 32144160 | rs277187 | A | 2.05E-03 | 2.61E-02 | 3.99E-04 | 9.76E-03 | 0.61 | 0.59 | 0.17 |
| 13 | 33663281 | rs9563143 | A | 3.95E-02 | 4.50E-03 | 4.66E-04 | 4.66E-04 | 1.78 | 1.78 | 0.80 |
| 13 | 41508851 | rs7329399 | T | 1.42E-03 | 3.54E-02 | 2.72E-04 | 1.60E-03 | 0.62 | 0.62 | 0.25 |
| 13 | 47794507 | rs11841008 | T | 3.64E-02 | 1.09E-03 | 1.15E-04 | 1.15E-04 | 1.51 | 1.51 | 0.66 |
| 13 | 49500173 | rs2770526 | G | 2.92E-04 | 2.71E-02 | 8.63E-05 | 1.12E-02 | 1.38 | 1.42 | 0.11 |
| 13 | 49930369 | rs7981156 | C | 8.83E-03 | 2.96E-02 | 9.03E-04 | 9.03E-04 | 0.77 | 0.77 | 0.45 |
| 13 | 50194394 | rs9535274 | A | 1.90E-02 | 1.90E-02 | 9.70E-04 | 9.70E-04 | 1.28 | 1.28 | 0.73 |
| 13 | 58765648 | rs9316978 | A | 3.06E-02 | 1.12E-02 | 8.81E-04 | 8.81E-04 | 1.31 | 1.31 | 0.88 |
| 13 | 78385158 | rs1759975 | A | 1.34E-02 | 1.49E-02 | 5.47E-04 | 5.47E-04 | 1.35 | 1.35 | 0.73 |
| 13 | 99417698 | rs7983023 | G | 1.27E-02 | 1.20E-02 | 4.53E-04 | 4.53E-04 | 0.77 | 0.77 | 0.64 |
| 13 | 110914530 | rs9559753 | T | 5.39E-03 | 8.47E-03 | 1.50E-04 | 1.50E-04 | 0.73 | 0.73 | 0.59 |
| 14 | 28803634 | rs8011268 | A | 1.62E-02 | 1.17E-02 | 5.17E-04 | 5.17E-04 | 0.60 | 0.60 | 0.77 |
| 14 | 52533443 | rs979481 | A | 5.79E-03 | 3.71E-02 | 8.03E-04 | 8.03E-04 | 0.75 | 0.75 | 0.39 |
| 14 | 68813083 | rs79383437 | A | 5.49E-03 | 7.67E-03 | 1.58E-04 | 1.58E-04 | 0.62 | 0.62 | 0.46 |
| 14 | 69331314 | rs56732174 | C | 2.62E-02 | 2.21E-03 | 1.59E-04 | 1.59E-04 | 1.44 | 1.44 | 0.83 |
| 14 | 77139517 | rs35787086 | T | 3.74E-02 | 4.16E-03 | 4.00E-04 | 4.00E-04 | 1.38 | 1.38 | 0.90 |
| 14 | 78379156 | rs17106604 | T | 5.13E-03 | 4.40E-02 | 8.25E-04 | 8.25E-04 | 1.35 | 1.35 | 0.40 |
| 14 | 85604943 | rs10130655 | G | 3.36E-02 | 5.82E-03 | 5.04E-04 | 5.04E-04 | 1.30 | 1.30 | 0.98 |
| 14 | 89788367 | rs11622771 | C | 6.38E-04 | 2.87E-02 | 1.52E-04 | 7.20E-03 | 0.73 | 0.72 | 0.15 |
| 14 | 89803269 | rs3783853 | A | 3.36E-03 | 2.83E-02 | 4.46E-04 | 7.67E-04 | 0.77 | 0.77 | 0.30 |
| 15 | 26366310 | rs17644708 | C | 9.51E-03 | 3.29E-03 | 9.19E-05 | 9.19E-05 | 1.38 | 1.38 | 0.76 |
| 15 | 34947425 | rs6495700 | G | 1.16E-02 | 4.87E-03 | 1.57E-04 | 1.57E-04 | 0.73 | 0.73 | 0.87 |
| 15 | 36424581 | rs16954322 | T | 2.59E-03 | 3.57E-02 | 4.19E-04 | 6.04E-04 | 1.35 | 1.35 | 0.30 |
| 15 | 45948198 | rs2670813 | C | 1.81E-02 | 1.64E-02 | 8.07E-04 | 8.07E-04 | 0.77 | 0.77 | 0.73 |
| 15 | 48083610 | rs9672207 | T | 1.33E-03 | 4.78E-02 | 4.43E-04 | 8.58E-03 | 1.32 | 1.34 | 0.17 |
| 15 | 52478297 | rs12903001 | T | 2.54E-02 | 7.64E-03 | 5.04E-04 | 5.04E-04 | 0.76 | 0.76 | 0.97 |
| 15 | 52512344 | rs79796987 | A | 1.87E-02 | 3.24E-03 | 1.65E-04 | 1.65E-04 | 1.52 | 1.52 | 0.97 |
| 15 | 54730873 | rs76139861 | A | 3.54E-04 | 2.37E-02 | 5.35E-05 | 1.07E-03 | 0.56 | 0.55 | 0.21 |
| 15 | 56316170 | rs35926005 | A | 7.52E-03 | 2.76E-02 | 7.85E-04 | 7.85E-04 | 0.75 | 0.75 | 0.40 |
| 15 | 80767141 | rs11852674 | G | 4.79E-03 | 2.86E-02 | 5.56E-04 | 5.56E-04 | 0.76 | 0.76 | 0.36 |
| 15 | 81813286 | rs17338496 | T | 3.45E-03 | 1.48E-02 | 1.92E-04 | 1.92E-04 | 0.68 | 0.68 | 0.45 |
| 15 | 91080553 | rs8040876 | G | 1.38E-02 | 4.09E-03 | 1.65E-04 | 1.65E-04 | 0.72 | 0.72 | 0.72 |
| 15 | 91098715 | rs10852133 | C | 5.38E-03 | 6.51E-04 | 1.09E-05 | 1.09E-05 | 0.69 | 0.69 | 0.88 |
| 15 | 95918789 | rs17667640 | C | 4.12E-03 | 4.22E-02 | 9.26E-04 | 4.93E-03 | 1.38 | 1.40 | 0.24 |
| 16 | 3214442 | rs2252009 | G | 1.27E-02 | 1.69E-02 | 6.21E-04 | 6.21E-04 | 1.29 | 1.29 | 0.64 |
| 16 | 3964751 | rs11076791 | C | 3.62E-04 | 7.73E-03 | 1.88E-05 | 4.45E-04 | 0.73 | 0.72 | 0.22 |
| 16 | 3987699 | rs7188043 | A | 3.31E-02 | 2.76E-04 | 3.17E-05 | 3.17E-05 | 1.42 | 1.42 | 0.50 |
| 16 | 6250908 | rs57029104 | A | 3.19E-04 | 3.89E-02 | 1.22E-04 | 1.12E-02 | 0.75 | 0.74 | 0.12 |
| 16 | 6252046 | rs62016040 | G | 2.88E-03 | 2.73E-02 | 4.05E-04 | 1.51E-03 | 1.34 | 1.35 | 0.26 |
| 16 | 6903222 | rs74007112 | T | 8.61E-03 | 1.32E-02 | 3.71E-04 | 3.71E-04 | 1.84 | 1.84 | 0.54 |
| 16 | 8830826 | rs4984999 | G | 3.99E-03 | 2.62E-02 | 4.25E-04 | 4.25E-04 | 1.30 | 1.30 | 0.36 |
| 16 | 17312099 | rs7202731 | A | 1.21E-02 | 1.35E-02 | 4.77E-04 | 4.77E-04 | 1.30 | 1.30 | 0.64 |
| 16 | 20144091 | rs1925518 | T | 8.47E-03 | 2.99E-02 | 8.11E-04 | 8.11E-04 | 1.59 | 1.59 | 0.52 |
| 16 | 26147666 | rs78103936 | A | 1.24E-03 | 9.01E-03 | 5.51E-05 | 5.51E-05 | 1.64 | 1.64 | 0.32 |
| 16 | 27432735 | rs2040790 | C | 1.63E-02 | 7.21E-03 | 3.25E-04 | 3.25E-04 | 1.30 | 1.30 | 0.80 |
| 16 | 31474418 | rs4262961 | A | 2.47E-02 | 1.22E-02 | 7.86E-04 | 7.86E-04 | 0.72 | 0.72 | 0.80 |
| 16 | 49769123 | rs116893108 | A | 3.38E-04 | 7.09E-03 | 1.36E-05 | 5.83E-05 | 2.06 | 2.08 | 0.28 |
| 16 | 52006578 | rs2160280 | C | 5.56E-03 | 3.38E-02 | 7.03E-04 | 7.03E-04 | 1.45 | 1.45 | 0.40 |
| 16 | 62002956 | rs11075447 | A | 1.46E-03 | 4.72E-02 | 3.35E-04 | 1.03E-03 | 0.68 | 0.68 | 0.27 |
| 16 | 73909538 | rs56971676 | C | 1.92E-04 | 4.45E-02 | 1.07E-04 | 1.82E-02 | 1.69 | 1.75 | 0.09 |
| 16 | 73981007 | rs12051297 | C | 7.61E-03 | 8.91E-03 | 2.13E-04 | 2.13E-04 | 1.33 | 1.33 | 0.63 |
| 16 | 80068414 | rs12597942 | T | 1.33E-02 | 1.67E-02 | 6.62E-04 | 6.62E-04 | 1.32 | 1.32 | 0.61 |
| 16 | 82497978 | rs7185396 | G | 8.10E-03 | 2.40E-02 | 6.46E-04 | 6.46E-04 | 0.77 | 0.77 | 0.49 |
| 16 | 86822348 | rs7199746 | A | 1.92E-03 | 1.59E-02 | 1.37E-04 | 1.37E-04 | 1.33 | 1.33 | 0.35 |
| 17 | 2863730 | rs9902973 | C | 5.36E-03 | 3.86E-02 | 8.10E-04 | 8.10E-04 | 1.29 | 1.29 | 0.37 |
| 17 | 2868935 | rs12950923 | T | 3.18E-03 | 2.59E-02 | 3.18E-04 | 3.18E-04 | 1.43 | 1.43 | 0.41 |
| 17 | 5698034 | rs758837 | T | 6.09E-03 | 1.13E-02 | 2.48E-04 | 2.48E-04 | 1.58 | 1.58 | 0.47 |
| 17 | 8675537 | rs2036171 | T | 7.19E-03 | 1.59E-02 | 4.00E-04 | 4.00E-04 | 0.70 | 0.70 | 0.48 |
| 17 | 8930080 | rs940850 | T | 4.41E-02 | 1.77E-03 | 2.17E-04 | 2.17E-04 | 0.74 | 0.74 | 0.70 |
| 17 | 8952894 | rs9915089 | T | 1.41E-03 | 2.33E-03 | 1.23E-05 | 1.23E-05 | 1.54 | 1.54 | 0.55 |
| 17 | 8977435 | rs12452924 | T | 5.73E-03 | 3.53E-02 | 7.68E-04 | 7.68E-04 | 1.34 | 1.34 | 0.39 |
| 17 | 8982132 | rs8079384 | A | 4.62E-03 | 1.86E-02 | 3.33E-04 | 3.33E-04 | 1.30 | 1.30 | 0.42 |
| 17 | 12307056 | rs381173 | C | 2.59E-02 | 1.15E-02 | 7.76E-04 | 7.76E-04 | 0.66 | 0.66 | 0.81 |
| 17 | 14717171 | rs7502709 | A | 7.03E-03 | 2.03E-02 | 5.12E-04 | 5.12E-04 | 0.67 | 0.67 | 0.45 |
| 17 | 32901461 | rs9916859 | A | 1.82E-02 | 1.87E-02 | 9.27E-04 | 9.27E-04 | 1.33 | 1.33 | 0.70 |
| 17 | 36032844 | rs1859212 | G | 8.89E-03 | 2.73E-02 | 7.99E-04 | 7.99E-04 | 0.79 | 0.79 | 0.49 |
| 17 | 48285769 | rs16970089 | A | 3.66E-03 | 4.04E-02 | 6.66E-04 | 9.98E-04 | 0.76 | 0.76 | 0.30 |
| 17 | 53608767 | rs1396215 | T | 2.92E-02 | 3.09E-03 | 2.42E-04 | 2.42E-04 | 1.33 | 1.33 | 0.94 |
| 17 | 54151310 | rs1431316 | T | 4.93E-02 | 7.75E-03 | 9.50E-04 | 9.50E-04 | 0.75 | 0.75 | 0.87 |
| 17 | 54773238 | rs227731 | G | 3.40E-02 | 8.47E-03 | 7.27E-04 | 7.27E-04 | 1.30 | 1.30 | 0.99 |
| 17 | 63739352 | rs12948946 | G | 4.86E-03 | 9.19E-03 | 1.55E-04 | 1.55E-04 | 0.58 | 0.58 | 0.52 |
| 17 | 64607335 | rs227915 | T | 2.24E-02 | 9.67E-03 | 5.64E-04 | 5.64E-04 | 1.38 | 1.38 | 0.91 |
| 17 | 72008083 | rs12951937 | A | 3.29E-03 | 2.30E-02 | 2.98E-04 | 2.98E-04 | 0.71 | 0.71 | 0.39 |
| 18 | 5878779 | rs12959856 | G | 2.82E-03 | 3.37E-02 | 5.24E-04 | 3.41E-03 | 1.33 | 1.35 | 0.23 |
| 18 | 7164754 | rs450015 | G | 3.77E-03 | 4.54E-02 | 7.23E-04 | 7.23E-04 | 1.36 | 1.36 | 0.33 |
| 18 | 7595095 | rs1941135 | A | 3.79E-02 | 3.43E-04 | 3.89E-05 | 3.89E-05 | 0.73 | 0.73 | 0.65 |
| 18 | 27403290 | rs16947191 | G | 3.50E-03 | 3.48E-02 | 5.55E-04 | 7.99E-04 | 1.30 | 1.30 | 0.30 |
| 18 | 31327296 | rs7243541 | C | 2.64E-03 | 2.35E-03 | 2.13E-05 | 2.13E-05 | 1.47 | 1.47 | 0.62 |
| 18 | 31406517 | rs73955054 | G | 2.52E-02 | 1.31E-02 | 8.44E-04 | 8.44E-04 | 1.71 | 1.71 | 0.91 |
| 18 | 48382636 | rs78458057 | C | 2.77E-02 | 5.09E-03 | 3.65E-04 | 3.65E-04 | 1.60 | 1.60 | 0.99 |
| 18 | 55267013 | rs4940928 | A | 2.85E-03 | 1.49E-02 | 1.82E-04 | 1.82E-04 | 0.74 | 0.74 | 0.36 |
| 18 | 60661222 | rs572638 | A | 1.58E-03 | 4.61E-02 | 4.12E-04 | 3.39E-03 | 1.30 | 1.31 | 0.22 |
| 18 | 74764925 | rs12607027 | A | 1.38E-02 | 1.19E-02 | 4.92E-04 | 4.92E-04 | 0.70 | 0.70 | 0.63 |
| 19 | 419407 | rs10408164 | A | 9.24E-04 | 4.25E-02 | 1.78E-04 | 2.12E-04 | 1.69 | 1.69 | 0.31 |
| 19 | 2534518 | rs10414848 | C | 1.13E-02 | 1.62E-02 | 5.52E-04 | 5.52E-04 | 1.41 | 1.41 | 0.61 |
| 19 | 3145887 | rs12461827 | T | 1.58E-02 | 4.72E-03 | 2.07E-04 | 2.07E-04 | 0.71 | 0.71 | 0.83 |
| 19 | 38732251 | rs111583246 | T | 3.35E-03 | 3.79E-02 | 6.05E-04 | 1.47E-03 | 1.36 | 1.37 | 0.28 |
| 19 | 51199571 | rs4802734 | C | 2.83E-03 | 4.85E-03 | 4.87E-05 | 4.87E-05 | 0.74 | 0.74 | 0.55 |
| 20 | 2205920 | rs6132441 | G | 3.42E-02 | 6.77E-03 | 5.85E-04 | 5.85E-04 | 0.77 | 0.77 | 0.97 |
| 20 | 5647738 | rs805751 | T | 1.59E-02 | 3.95E-03 | 1.72E-04 | 1.72E-04 | 1.39 | 1.39 | 0.97 |
| 20 | 8269273 | rs6077342 | A | 1.95E-03 | 3.06E-02 | 3.15E-04 | 1.44E-03 | 0.77 | 0.76 | 0.26 |
| 20 | 11764431 | rs2249353 | G | 4.27E-02 | 6.84E-03 | 7.30E-04 | 7.30E-04 | 1.29 | 1.29 | 0.98 |
| 20 | 14333855 | rs6079396 | G | 1.18E-02 | 1.36E-02 | 4.77E-04 | 4.77E-04 | 1.41 | 1.41 | 0.63 |
| 20 | 40611688 | rs6093544 | T | 5.43E-03 | 8.07E-03 | 1.47E-04 | 1.47E-04 | 0.74 | 0.74 | 0.56 |
| 20 | 40739308 | rs45543236 | A | 3.73E-02 | 3.13E-03 | 3.08E-04 | 3.08E-04 | 0.65 | 0.65 | 0.83 |
| 20 | 42454828 | rs34595395 | C | 9.43E-03 | 5.67E-03 | 1.54E-04 | 1.54E-04 | 0.74 | 0.74 | 0.81 |
| 20 | 43874078 | rs12481455 | C | 4.58E-02 | 2.72E-03 | 3.76E-04 | 3.76E-04 | 1.70 | 1.70 | 0.57 |
| 20 | 44085460 | rs1016496 | G | 1.03E-02 | 2.65E-02 | 8.93E-04 | 8.93E-04 | 1.27 | 1.27 | 0.50 |
| 20 | 44114873 | rs6065823 | C | 1.68E-02 | 2.14E-02 | 9.94E-04 | 9.94E-04 | 0.78 | 0.78 | 0.67 |
| 20 | 48407680 | rs11905002 | A | 4.83E-03 | 2.87E-02 | 5.46E-04 | 5.46E-04 | 1.31 | 1.31 | 0.38 |
| 21 | 27335022 | rs2014146 | G | 3.54E-02 | 3.38E-03 | 3.22E-04 | 3.22E-04 | 1.32 | 1.32 | 0.79 |
| 21 | 44145135 | rs2284963 | T | 3.87E-02 | 1.04E-02 | 9.94E-04 | 9.94E-04 | 0.77 | 0.77 | 0.95 |
| 22 | 17280822 | rs5748648 | A | 2.04E-02 | 1.79E-02 | 9.96E-04 | 9.96E-04 | 0.67 | 0.67 | 0.70 |
| 22 | 19364244 | rs17811102 | T | 1.14E-02 | 2.40E-02 | 7.50E-04 | 7.50E-04 | 1.43 | 1.43 | 0.72 |
| 22 | 19913093 | rs5993871 | G | 2.17E-02 | 1.65E-02 | 9.41E-04 | 9.41E-04 | 1.31 | 1.31 | 0.79 |
| 22 | 21943938 | rs1034329 | C | 4.06E-03 | 4.15E-02 | 7.13E-04 | 7.13E-04 | 0.79 | 0.79 | 0.33 |
| 22 | 25110395 | rs140347 | T | 8.57E-04 | 2.93E-02 | 1.75E-04 | 4.28E-03 | 0.74 | 0.73 | 0.18 |
| 22 | 27546776 | rs5997182 | G | 1.92E-02 | 1.57E-02 | 8.11E-04 | 8.11E-04 | 1.28 | 1.28 | 0.74 |
| 22 | 27580688 | rs9613393 | G | 1.16E-02 | 7.87E-03 | 2.52E-04 | 2.52E-04 | 1.63 | 1.63 | 0.82 |
| 22 | 28066600 | rs134085 | G | 1.80E-03 | 2.91E-02 | 2.58E-04 | 6.44E-04 | 1.64 | 1.65 | 0.28 |
| 22 | 36104647 | rs4820209 | A | 2.56E-02 | 1.26E-02 | 8.20E-04 | 8.20E-04 | 1.30 | 1.30 | 0.88 |
| 22 | 40240499 | rs6001694 | G | 2.11E-02 | 1.01E-02 | 5.56E-04 | 5.56E-04 | 0.66 | 0.66 | 0.89 |
| 22 | 47153559 | rs801646 | T | 7.08E-03 | 1.32E-02 | 3.10E-04 | 3.10E-04 | 1.30 | 1.30 | 0.53 |
| 22 | 49573505 | rs2318942 | A | 4.73E-04 | 1.44E-02 | 4.60E-05 | 1.16E-03 | 0.72 | 0.71 | 0.21 |
| 22 | 50092148 | rs738413 | C | 1.24E-02 | 6.23E-03 | 2.24E-04 | 2.24E-04 | 0.75 | 0.75 | 0.74 |

| chr | pos (hg38) | variant | Ref | Alt | SiPhy cons | Promoter histone marks | Enhancer histone marks | DNAse | Proteins bound | Motifs changed | NHGRI/EBI GWAS hits | GRASP QTL hits | Selected eQTL hits | GENCODE genes | dbSNP func annot |
| --- | --- | --- | --- | --- | --- | --- | --- | --- | --- | --- | --- | --- | --- | --- | --- |
|  |  |  |  |  |  |  |  |  |  |  |  |  |  |  |  |
| 1 | 210224051 | rs11119445 | G | A |  |  | KID, GI |  |  | TEF-1 |  |  |  | 7.4kb 3' of *C1orf133* |  |
| 1 | 210021655 | rs227227 | C | T |  |  | BRN | BRN,BRN |  | Hbp1,YY1,  ZEB1 |  |  |  | *SYT14* | intronic |
| 1 | 210042069 | rs12561877 | C | T |  |  |  |  |  | TEF-1 |  |  |  | *SYT14* | intronic |
| 1 | 209761496 | rs643118 | T | C |  |  | 5 tissues | 4 tissues |  |  |  | 1 hit | 8 hits | *TRAF3IP3* | intronic |
| 9 | 124528833 | rs2095293 | C | T |  |  | ADRL |  |  | Pou2f2,  Pou4f3,  Pou6f1 |  |  |  | *NR6A1* | intronic |

**Supplementary Table 3.** Function annotation for the five newly identified SNPs in Haploreg v4.1

| **Supplementary Table 4.** The linkage disequilibrium (LD) between newly identified SNPs in the current study and other previously established SNPs in this region reported to be associated with NSCL/P in Asian (CHB+JPT) populations using data from the 1,000 Genomes Project | | | | | | | |
| --- | --- | --- | --- | --- | --- | --- | --- |
| Cytoband | BP | Novel SNPs | BP | Previously established SNPs | r^2^ | GWAS target | Reference |
| 1q32.2 | 210397396 | rs11119445 | 209964080 | rs2235371 | 0.27 | Nonsyndromic cleft lip with or without cleft palate | Sun |
| 1q32.2 |  |  | 209977111 | rs861020 | 0.06 | Nonsyndromic cleft lip with or without cleft palate | Ludwig |
| 1q32.2 |  |  | 209982025 | rs6540559 | 0.23 | Cleft lip with or without cleft palate | Leslie |
| 1q32.2 |  |  | 209984470 | rs75477785 | 0.27 | Cleft lip with or without cleft palate | Leslie |
| 1q32.2 |  |  | 209988047 | rs10863790 | 0.27 | Cleft lip | Beaty |
| 1q32.2 |  |  | 209989270 | rs642961 | 0.06 | Orofacial clefts | Birnbaum |
| 1q32.2 |  |  | 210048819 | rs2064163 | 0.46 | Nonsyndromic cleft lip with cleft palate | Yu |
| 1q32.2 |  |  | 210050794 | rs9430019 | 0.11 | Nonsyndromic cleft lip with cleft palate | Yu |
| 1q32.2 | 210195000 | rs227227 | 209964080 | rs2235371 | 0.28 | Nonsyndromic cleft lip with or without cleft palate | Sun |
| 1q32.2 |  |  | 209977111 | rs861020 | 0.12 | Nonsyndromic cleft lip with or without cleft palate | Ludwig |
| 1q32.2 |  |  | 209982025 | rs6540559 | 0.20 | Cleft lip with or without cleft palate | Leslie |
| 1q32.2 |  |  | 209984470 | rs75477785 | 0.28 | Cleft lip with or without cleft palate | Leslie |
| 1q32.2 |  |  | 209988047 | rs10863790 | 0.28 | Cleft lip | Beaty |
| 1q32.2 |  |  | 209989270 | rs642961 | 0.12 | Orofacial clefts | Birnbaum |
| 1q32.2 |  |  | 210048819 | rs2064163 | 0.29 | Nonsyndromic cleft lip with cleft palate | Yu |
| 1q32.2 |  |  | 210050794 | rs9430019 | 0.13 | Nonsyndromic cleft lip with cleft palate | Yu |
| 1q32.2 | 210215414 | rs12561877 | 209964080 | rs2235371 | 0.23 | Nonsyndromic cleft lip with or without cleft palate | Sun |
| 1q32.2 |  |  | 209977111 | rs861020 | 0.05 | Nonsyndromic cleft lip with or without cleft palate | Ludwig |
| 1q32.2 |  |  | 209982025 | rs6540559 | 0.19 | Cleft lip with or without cleft palate | Leslie |
| 1q32.2 |  |  | 209984470 | rs75477785 | 0.23 | Cleft lip with or without cleft palate | Leslie |
| 1q32.2 |  |  | 209988047 | rs10863790 | 0.23 | Cleft lip | Beaty |
| 1q32.2 |  |  | 209989270 | rs642961 | 0.05 | Orofacial clefts | Birnbaum |
| 1q32.2 |  |  | 210048819 | rs2064163 | 0.42 | Nonsyndromic cleft lip with cleft palate | Yu |
| 1q32.2 |  |  | 210050794 | rs9430019 | 0.11 | Nonsyndromic cleft lip with cleft palate | Yu |
| 1q32.2 | 209761496 | rs643118 | 209964080 | rs2235371 | 0.04 | Nonsyndromic cleft lip with or without cleft palate | Sun |
| 1q32.2 |  |  | 209977111 | rs861020 | 0.32 | Nonsyndromic cleft lip with or without cleft palate | Ludwig |
| 1q32.2 |  |  | 209982025 | rs6540559 | 0.03 | Cleft lip with or without cleft palate | Leslie |
| 1q32.2 |  |  | 209984470 | rs75477785 | 0.04 | Cleft lip with or without cleft palate | Leslie |
| 1q32.2 |  |  | 209988047 | rs10863790 | 0.04 | Cleft lip | Beaty |
| 1q32.2 |  |  | 209989270 | rs642961 | 0.33 | Orofacial clefts | Birnbaum |
| 1q32.2 |  |  | 210048819 | rs2064163 | 0.03 | Nonsyndromic cleft lip with cleft palate | Yu |
| 1q32.2 |  |  | 210050794 | rs9430019 | 0.03 | Nonsyndromic cleft lip with cleft palate | Yu |
